# Mitigation of COVID-19 through onsite testing and education among formerly incarcerated individuals (the MOSAIC study): an open-label, single-centre, randomised controlled trial

**DOI:** 10.1016/S2468-2667(26)00093-9

**Published:** 2026-07

**Authors:** Matthew J Akiyama, Fatoumata Kaba-Diakite, Mariah Dimaulaluan, Lindsey R Riback, Jordy Rojas Antigua, Connor S Holmes, Maxwell F Ackerman, Amour Castillo, Micaela Linder, Ronald F Day, Ann Travers, Chinazo O Cunningham, Yuting Deng, Chenshu Zhang, Aaron D Fox

**Affiliations:** Albert Einstein College of Medicine, Montefiore Medical Center, Bronx, NY, USA (M J Akiyama MD, F Kaba-Diakite MPH, M Dimaulaluan BA, L R Riback MPH, J R Antigua BS, C S Holmes BS, M F Ackerman BS, Prof C O Cunningham MD, Y Deng MPH, C Zhang PhD, Prof A D Fox MD); The Fortune Society, Long Island City, NY, USA (A Castillo MPhil, M Linder MPhil, R F Day PhD, A Travers BS)

## Abstract

**Background:**

Criminal legal system-involved individuals face barriers to care after release from carceral settings. Elevated SARS-CoV-2 risk while incarcerated, together with living in congregate settings post-incarceration, increase the risk of respiratory viral infection transmission. This study evaluated a community health worker-led point-of-care SARS-CoV-2 testing and education intervention in a re-entry-focused community-based organisation compared with standard referrals.

**Methods:**

This non-blinded, parallel group, randomised controlled trial, conducted in partnership with a community-based organisation in New York City (NYC), NY, USA, enrolled clients who were released from incarceration in the previous 90 days, fluent in English or Spanish, and residing within NYC for the study duration. Participants were randomly assigned (1:1) via computer-generated randomisation to onsite point-of-care testing and education or standard of care referral to offsite testing sites over 12 months. All participants were advised to test every 3 months. The study was not masked due to the nature of the intervention. The primary outcome was the proportion of patients with at least one SARS-CoV-2 test performed with results received during the 12-month period. Primary analyses were done by intention-to-treat using logistic or Poisson regression modelling. This trial was registered with ClinicalTrials.gov, NCT04878328, and is completed.

**Findings:**

Between April 14, 2022, and May 22, 2024, 572 formerly incarcerated individuals were assessed for study eligibility. After the exclusion of 247 individuals, and a further 75 who did not attend enrolment, 250 participants were randomly assigned to the two study groups (125 to onsite point-of-care testing and education and 125 to standard of care). 216 (86%) participants were cisgender men, 30 (12%) were cisgender women, two (1%) were transgender women, and two (1%) were non-binary; 120 (48%) were Black and 82 (33%) were Hispanic; mean age was 42·0 years (SD 11·8). 109 (87%) of the 125 participants in the onsite point-of-care testing and education group and 67 (54%) of 125 participants in the standard of care group had at least one complete SARS-CoV-2 test (odds ratio 5·9 [95% CI 3·1–11·1]; p<0·0001). The absolute difference was 34 percentage points (95% CI 23–44; p<0·0001). The incidence rate ratio of complete SARS-CoV-2 tests was 2·4 times (95% CI 1·9–3·0, p<0·0001) as high among participants in the intervention group versus those in the standard of care group. No serious adverse events occurred.

**Interpretation:**

Community-health worker-led testing and education at a re-entry-focused community-based organisation could potentially increase uptake of SARS-CoV-2 testing among formerly incarcerated individuals. Although further, larger trials are required, trusted community health workers can provide onsite point-of-care testing and health education, which has relevance for respiratory viral infections such as SARS-CoV-2 and might potentially be applicable to other infectious diseases such as HIV and hepatitis C virus.

**Funding:**

US National Institutes of Health.

## Introduction

Carceral institutions have facilitated the spread of respiratory infectious diseases, such as SARS-CoV-2, given the congregate nature of their living arrangements and the tendency towards overcrowding.^1^ Among 53 US prison systems assessed up to July 15, 2020, the SARS-CoV-2 positivity rate was higher on average than in the general population, with rates as high as 42%, and many of the largest single-site US SARS-CoV-2 outbreaks occurred in prisons.^2^ Internationally, some carceral systems attempted to mitigate the effect of the COVID-19 pandemic through decarceration, but many US carceral facilities faced operational challenges in implementing public health measures.^3^ Because 95% of formerly incarcerated individuals return to their communities, elevated SARS-CoV-2 risk while incarcerated, coupled with the high likelihood of living in congregate settings (eg, shelters) after incarceration,^4^ creates high-risk conditions for SARS-CoV-2 transmission. Yet, testing procedures are inconsistent within and across carceral facilities,^5^ particularly during the early stages of the COVID-19 pandemic.^6,7^ Recommendations regarding testing these individuals as they transition to the community, a process that is often rushed and without adequate discharge planning, are even less consistent.^8^ SARS-CoV-2 and other highly transmissible respiratory pathogens such as tuberculosis, influenza, and measles, during incarceration and upon return to the community, are of ongoing concern;^9–11^ however, the disproportionate impact of the COVID-19 pandemic among incarcerated individuals was an unprecedented public health threat.^1^

Targeting prevention and mitigation strategies, such as screening, wearing of face masks, and social distancing, to the post-incarceration period could be beneficial during respiratory viral pandemics. High rates of unstable housing and reincarceration can lead to transmission in congregate housing and reintroduction of SARS-CoV-2 to prisons upon reincarceration.^4^ In a multisite US study conducted by the Transitions Clinic Network, among 751 participants who were linked soon after release from prison to specialised primary care practices, 24·6% reported being homeless and living in shelters; 37·5% reported living in transitional housing (including drug treatment and halfway houses); 27·8% reported being doubled up with friends or family; and only 10% rented or owned their own apartment or house.^4^ Screening reveals whether someone is a SARS-CoV-2 carrier and could help to prevent transmission in these settings if coupled with changes in behaviour. Masking and social distancing, the non-pharmacological interventions with the largest evidence base early in the pandemic, allow for the interruption of forward transmission and acquisition of infection.^12,13^ Yet to our knowledge, there are no high-quality randomised controlled trials of SARS-CoV-2 testing and mitigation strategies among formerly incarcerated individuals. Uptake of public health interventions among this population might be particularly challenging due to medical mistrust,^7,14^ limited access to testing or testing fatigue,^7^ limited access to personal protective equipment,^5,14^ and difficulty social distancing while residing in congregate housing.^5,15^

Some data from low-resource settings and minority populations in the USA suggest that community health worker-led strategies increased SARS-CoV-2 testing uptake compared with existing standards of care.^16,17^ Community health workers receive training in health education and health system navigation and often share important characteristics, such as previous incarceration, with the communities they serve.^18^ In San Diego, the LinkUP intervention provided tailored education, motivational interviewing, and planning delivered by trained peer counsellors to increase COVID-19 testing uptake among people who inject drugs.^19^ Although these studies demonstrate promise for community health worker or peer interventions to improve SARS-CoV-2 testing among marginalised populations, these studies have not focused on formerly incarcerated individuals, the effect of community health worker education on adherence to mitigation behaviours, or the effect of co-located COVID-19 services within a community-based organisation focused on formerly incarcerated individuals.

Reentry-focused community-based organisations provide important services, such as employment training, housing assistance, and linkage to medical care, for people released from carceral settings. During the COVID-19 pandemic, several community-based organisations provided SARS-CoV-2 testing^20^ and vaccine education.^21^ Community health workers can allow community-based organisations to expand health-care delivery.^22–26^ For formerly incarcerated individuals, community health workers circumvent barriers, such as medical mistrust and stigma,^27^ to build trust, provide support and health education, and link this population to health-care services.^28,29^ For example, in a randomised controlled trial involving people with HIV leaving Los Angeles County Jail, the LINK-LA study demonstrated peer navigation was successful at preventing declines in viral suppression, typically seen after release from incarceration, compared with standard transitional case management.^30^

The mitigation through onsite testing and education among formerly incarcerated individuals against COVID-19 (MOSAIC) study’s goal was to examine a SARS-CoV-2 mitigation strategy in which a trained community health worker provided point-of-care testing and education at a re-entry-focused community-based organisation. In this study, we investigated whether this strategy could promote positive COVID-19 health outcomes among formerly incarcerated individuals. We hypothesised that our intervention would improve testing uptake and receipt of test results by offering onsite care at the community-based organisation and adherence to mitigation behaviours through community health worker-delivered education. We did not anticipate any harms.

## Methods

### Study design

In this non-blinded, parallel-group, randomised controlled trial, we compared a community health worker-delivered point-of-care testing and education intervention onsite at a community-based organisation with standard of care (referral to offsite community testing locations) among formerly incarcerated individuals. All participants were advised to test every 3 months according to their study group, and were followed up over 12 months. Trial planning began in the latter half of 2020; funding was awarded in December, 2021; and enrolment began on April 14, 2022.

The trial was conducted in partnership with The Fortune Society (Fortune), a community-based organisation in New York City (NYC), NY, USA, that serves about 18 000 formerly incarcerated individuals per year returning from jail or prison, with the mission of supporting successful re-entry and promoting alternatives to incarceration.^31,32^ Fortune’s services include assistance with health insurance, benefit enrolment, education, and employment services. Fortune operates seven sites in NYC and has 581 employees. The community-based organisation’s client population (90% male, 50% Black, 24% Latinx) is reflective of the general NYC population of formerly incarcerated individuals. Study procedures, including recruitment, occurred at two of Fortune’s main sites. The research team was primarily based in one service site, but participants randomly assigned to onsite point-of-care testing and education were informed that they could complete community health worker testing and education visits at the second location if they preferred.

This trial was registered with ClinicalTrials.gov, NCT04878328, and approved by Albert Einstein College of Medicine’s Institutional Review Board under protocol 2021–12976 and completed on May 25, 2025. A Data and Safety Monitoring Board approved the protocol and monitored study progress every 6 months or as deemed necessary. Participants were not involved in the study design, conduct, or reporting. The protocol is provided in appendix 1.

### Participants

We recruited adult clients of Fortune who had returned from incarceration within the past 90 days. Participants were fluent in English or Spanish and planned to reside within NYC for the duration of the study.^33^ Individuals were excluded if they were unable to provide informed consent, could not complete study visits over 12 months, did not plan to reside in the NYC area for the year following enrolment, or had a terminal illness. Fortune staff provided referrals to the research team and study flyers were posted at Fortune locations. Research assistants (CSH, FK-D, JRA, LRR, MFA, and MD) screened interested clients over the telephone or in person and determined release dates in public databases. Research assistants (CSH, FK-D, JRA LRR, MFA, and MD) scheduled an in-person enrolment visit for eligible participants during which they described the study and conducted informed consent procedures. Potential participants reviewed informed consent forms, had the opportunity to ask questions about the study, and provided written consent. Once informed consent was obtained, the research assistant (CSH, FK-D, JRA, LRR, MFA, and MD) proceeded with the baseline survey, followed by randomisation.

All survey data were collected via self-report. Race and ethnicity data were collected through the demographics form. We asked “How would you describe your race or ethnicity?” The options were White, Black or African American, Native American or Alaska Native, Chinese, Filipino, Asian Indian, Vietnamese, Korean, Japanese, Other Asian, Native Hawaiian, Samoan, Chamorro, Other Pacific Islander, Some other race, Don’t know, and Refuse to answer. They were then collapsed to Non-Hispanic Black, Hispanic, Non-Hispanic White, and Non-Hispanic Other. Gender data were also collected via the demographics form: “What terms best express how you describe your gender identity?” The choices were Man, Woman, Non-binary, Transgender, None of these describe me and I would like additional options, or Prefer not to answer. Transgender was updated to Transgender female because two participants identified as women.

### Randomisation and masking

Once the enrolment visit was completed, the research assistant (CSH, FK-D, JRA, LRR, MFA, and MD) randomly assigned eligible participants (1:1; blocks of four to eight) to onsite point-of-care testing and education or standard of care via computer-generated randomisation, programmed by a biostatistician (CZ) in SAS (version 9.4) into REDCap. Masking was not possible because the intervention was an interactive behavioural programme that required active engagement from both participants and implementers.

### Procedures

Participants in both groups were contacted by telephone within 1 week of randomisation to receive education about SARS-CoV-2 testing, including the role of asymptomatic testing. Participants were advised to test every 3 months for 1 year regardless of their last test result, and received reminders about testing visits at equivalent intervals. Testing in the onsite point-of-care group was conducted at Fortune, and testing in the standard of care group was via referral to a community testing location. Participants were not compensated for testing. During the trial, participants, regardless of the group to which they were assigned, had full access to Fortune services.

Our intervention, which combined onsite SARS-CoV-2 testing at Fortune and community health worker support, was co-designed in partnership with the leadership and staff of Fortune. We recruited one community health worker with lived experience of incarceration who was a member of the Fortune community to provide SARS-CoV-2 testing, education, and navigation services. The community health worker had previous experience providing COVID-19 education and distributing masks through a previous role with NYC Test and Trace Corps. They were selected due to their commitment to working with formerly incarcerated individuals and their effective communication skills. In addition to the standard procedural training, the research team provided training on principles of the Health Insurance Portability and Accountability Act confidentiality and accessing updated information on COVID-19 prevention, testing, and vaccination sites in NYC.^34^ The community health worker provided: COVID-19 education; onsite SARS-CoV-2 PCR testing at Fortune facilities; needs assessments for unmet social needs (adapted from Fortune’s intake form); access to face masks and hygiene supplies; navigation to vaccination sites; and general psychosocial support. Community health worker-led education was based on the best available information in the literature and evidence-informed public health guidelines. Participants in the onsite point-of-care testing and education group could choose between two of the community-based organisation offices in different NYC boroughs for their visits with the community health worker.

Cepheid GeneXpert Xpress System SARS-CoV-2 PCR testing equipment (Sunnyvale, CA, USA) was used for SARS-CoV-2 testing, and the community health worker received training on specimen handling and quality control. All equipment was provided by Cepheid and stored in the community health worker’s private offices. GeneXpert results are available in approximately 30 min. While awaiting results, the community health worker delivered the other intervention components. If participants tested positive on the PCR, the community health worker provided guidance on social distancing and referral to clinical care when indicated.

Participants in the standard of care group did not meet with the community health worker for testing and education. They instead worked with the project coordinator (AC) at Fortune to find a convenient offsite testing location using the NYC COVID-19 Test Site Finder.^35^ This included community health sites, hospitals, urgent care centres, and street vendors. This referral protocol was developed from the standard referral process Fortune case managers use for other services including health care. Referrals for SARS-CoV-2 testing every 3 months in the standard of care group was above the standard of care provided at Fortune, which included assistance with locating testing sites as needed.

Data sources included quarterly biobehavioural questionnaires, biweekly brief smartphone surveys, and Fortune programme logs. The quarterly biobehavioural questionnaires were administered by research assistants (CSH, FK-D, JRA, LRR, MFA, and MD) virtually or in an onsite study room, based on participants’ availability, using REDCap, a secure, web-based data capture software platform. Questionnaires included key sociodemographic, comorbidity (eg, mental health), and COVID-19-related variables, all self-reported as previously described.^33^ Participants were compensated US$50 for quarterly visits, with an additional $5·80 for in-person visits to compensate for travel (a return trip NYC metrocard). To improve retention efforts, starting in March, 2024, participants were compensated an additional $50 if they completed both the month 3 and month 6 visit, and another $50 if they completed both the month 9 and the month 12 visit.

To improve measurement of adherence to mitigation behaviours and minimise recall bias, participants also completed brief smartphone surveys using a mobile application on pre-paid study smartphones every 2 weeks for 12 months (26 total surveys). Surveys took 5–10 min to complete and included questions about participants’ testing and mitigation behaviours over the past 2 weeks (eg, use of face masks, ability to socially distance, and other protective measures). Participants were compensated $10 for each of the surveys they completed. Community health worker visits and testing uptake for the onsite point-of-care testing and education group, and referrals made and testing uptake for the standard of care group, were all recorded in programme logs.

### Outcomes

The primary objective was to test onsite point-of-care testing and education versus standard of care regarding the proportion of participants who completed and received results for at least one SARS-CoV-2 test during the 12-month study period. To measure testing uptake, we used a dichotomous outcome of having at least one complete SARS-CoV-2 test over the 12-month study period, defined as having a test performed with results received by participants within 2 weeks of the test being conducted. We chose the composite measure of tests performed and results received because having test results would aid participants in adjusting their mitigation behaviours.

The secondary objective was to determine adherence to mitigation measures such as face-mask use, social distancing, and handwashing. We measured adherence to mitigation measures from the biweekly surveys. Masking behaviour was measured using a composite weighted variable estimating how frequently in the past 2 weeks participants went to places where they could have been exposed to COVID-19 and how often they wore a mask during these activities. Initial responses of 1, always; 2, most of the time; 3, sometimes; 4, rarely; or 5, never, were standardised to a scale of 1–100. We measured the participants’ social distancing behaviour by their responses to the following question: overall, on a scale of 1–100, how often did you stay greater than 6 feet away from others when you were not masked in the past 2 weeks? Participants’ handwashing behaviour was measured by the following question: overall, on a scale of 1–100, how often did you wash hands after shaking hands with or touching surfaces that were touched by someone else in the past 2 weeks? A higher score reflected more consistent application of a mitigation behaviour.

Research assistants (CSH, FK-D, JRA, LRR, MFA, and MD) captured any reported adverse events during scheduled research visits. Because risks of SARS-CoV-2 testing, education, and navigation activities were commensurate with those of routine clinical care, there was not a systematic assessment for adverse events. Those that occurred were assessed clinically and followed to resolution. The severity of adverse events was categorised according to institutional review board regulations. All adverse events were recorded and are documented in appendix 2 (p 3).

### Statistical analysis

We designed our study to enrol 125 participants per group, conservatively accounting for a 15% attrition. The sample size was estimated to provide sufficient power to detect statistically significant differences in testing uptake and receipt (primary outcome) and mitigation measures (secondary outcome). For our primary outcome, this would have allowed us to detect a 19 percentage point difference (70% *vs* 51%) between groups in the proportion of participants with at least one test performed and results received with at least 80% of power at a significance level of 5% (two-sided test). This effect size was comparable to that observed in a study of onsite, point-of-care testing for HIV and hepatitis C virus (HCV) in a substance use treatment programme (*vs* offsite referral).^36^ The sample size also allowed sufficient power for planned secondary analyses.^33^

We first examined baseline characteristics and histories of COVID-19 tests, SARS-CoV-2 infections, and COVID-19 vaccinations by study group. Primary analyses were done by intention-to-treat, meaning that all enrolled participants were analysed according to the groups to which they were originally assigned, regardless of the intervention they received or whether they completed full study follow-up. We also conducted per-protocol analyses excluding participants who had not received a testing referral. Participants missing primary outcome data were assumed to have not completed study-related SARS-CoV-2 testing. Interim analyses were conducted annually as part of Data and Safety Monitoring Board proceedings (appendix 2 pp 5–8).

In our primary analysis, for each quarter (0–3 months, 3–6 months, 6–9 months, and 9–12 months), an indicator variable for the status of complete SARS-CoV-2 testing (yes or no) was generated. Based on the quarterly records, we determined whether participants had at least one complete SARS-CoV-2 test for the whole 12-month study period and the total number of complete SARS-CoV-2 tests (up to five tests). We conducted logistic regression with study group as the main independent variable and having at least one complete SARS-CoV-2 test during the 12-month study period as the dependent variable to estimate odds ratios. We also conducted Poisson regression models as co-primary analyses to compare the total number of SARS-CoV-2 tests performed and the number of SARS-CoV-2 test results received between groups during the 12-month study period. The incidence rate ratios were calculated based on mean number of tests over 12 months. Additionally, we evaluated the between-group difference for the primary outcome by calculating absolute difference in proportion, expressed as percentage points, with corresponding 95% CIs.

Secondary analyses were conducted using generalised estimating equations (SAS Proc Genmod), which allowed us to analyse correlated data. These analyses examined the associations between: (1) onsite point-of-care testing and education (versus standard of care) and adherence to mitigation measures; and (2) onsite point-of-care testing and education intervention dose (ie, the number of testing and education visits with the community health worker) and subsequent adherence to mitigation measures (onsite point-of-care testing and education group only). Participants’ 26 brief surveys assessing mitigation measures over 2-week intervals were the unit of analysis. In the first model, the primary dependent variable was each of the mitigation measures and the main independent variable was randomisation group. In the second model, the independent variable was a time-varying indicator variable of onsite point-of-care testing and education attendance status, which was assigned a score of 1 when a smartphone survey date was within 90 days of an onsite point-of-care testing and education visit attendance date, and 0 otherwise. The dependent variable was each of the mitigation measures.

For each measure of our secondary outcome variables (continuous), we first used data from non-missing visits and then conducted multiple imputations using Markov Chain Monte Carlo imputation methods for missing data. Based on the method proposed by Rubin,^37^ we pooled analytic results from the multiple imputed datasets into a single result. All analyses were done with SAS (version 9.4).

### Role of the funding source

The funder of the study had no role in study design, data collection, data analysis, data interpretation, or writing of the report.

## Results

572 people were assessed for eligibility, of whom 247 were excluded and a further 75 did not attend enrolment. 250 participants were enrolled in the study from April 14, 2022, to May 22, 2024, and randomly assigned to the two study groups (125 to onsite point-of-care testing and education and 125 to standard of care; figure). Adverse events were recorded and are documented in appendix 2 (p 3). The final participant completed the study protocol on May 25, 2025. We report baseline characteristics by study group in table 1. 216 (86%) participants were cisgender men, 30 (12%) were cisgender women, two (1%) were transgender women, and two (1%) were non-binary; 120 (48%) were Black and 82 (33%) were Hispanic; and 173 (69%) reported living in congregate settings at enrolment. The mean age of participants was 42·0 years (SD 11·8), and mean number of years incarcerated in lifetime was 12·8 years (11·6). Before enrolment, 239 (96%) had reported ever being tested for COVID-19 and 85 (34%) reported a previous positive test. Most participants (192 [77%]) had received at least one COVID-19 vaccine dose, with 95 (38%) having received at least one booster.

During the 12-month study period, 109 (87%) of the 125 participants in the onsite point-of-care testing and education group and 67 (54%) of 125 participants in the standard of care group had at least one complete SARS-CoV-2 test. The absolute difference was 34 percentage points (95% CI 23–44; p<0·0001). In the onsite point-of-care testing and education group, 101 participants had at least one complete test at baseline, 67 at 3 months, 54 at 6 months, 37 at 9 months, and 34 at 12 months. In the standard of care group, 61 participants had at least one complete test at baseline, 30 at 3 months, 11 at 6 months, 14 at 9 months, and eight at 12 months. Participants in the intervention group were more likely to have at least one complete SARS-CoV-2 test during the 12-month study period (odds ratio 5·9 (95% CI 3·1–11·1; p<0·0001) than were participants in the standard of care group. The mean number of completed tests over 12 months was 2·3 (SD 1·6) in the intervention group and 1·0 (1·2) in the standard of care group. Results of the bivariate Poisson regression analysis indicated that the incidence rate ratio (IRR) of complete SARS-CoV-2 tests was 2·4 times (95% CI 1·9–3·0; p<0·0001) higher in the intervention group than in the standard of care group (table 2). The per-protocol analysis did not substantially change these results (appendix 2 p 2).

Among the 250 participants, 229 (92%) reported mitigation behaviours in at least one biweekly smartphone survey (115 in the onsite point-of-care testing and education group and 114 in the standard of care group). 32 (14%) of the 229 participants completed all 26 web-surveys. The mean number of completed web-surveys was 16·1 (SD 8·6). There was no significant difference in the mean number of completed web-surveys (mean difference 1·5 [95% CI −0·8 to 3·7]; p=0·21) between the onsite point-of-care testing and education group (mean 16·9 [SD 8·3]) and the standard of care group (15·4 [8·9]). On a scale of 1–100, the mean adherence masking score was 41·3 (SD 35·8), social distancing was 52·6 (35·4), and handwashing was 72·1 (30·7), for all participant visits. Among participants in the intervention group, the mean adherence scores of the mitigation behaviour measures were 45·1 (SD 35·8) for masking, 55·3 (35·3) for social distancing, and 73·8 (29·9) for handwashing. Among participants in the standard of care group, the scores were 37·0 (SD 35·3) for masking, 49·6 (35·4) for social distancing, and 70·3 (31·5) for handwashing.

Results of generalised estimating equations models for the associations between study groups and mitigation behaviours over the 12-month study period while accounting for individual-level correlations are presented in table 2. For these analyses, β represents the average change in the outcome measure on a scale from 1–100. First, we excluded observations with missing data on the dependent variable in the analysis. Compared with the standard of care group, participants in the onsite point-of-care testing and education group had significantly greater adherence to masking behaviours (β 8·44 [95% CI 0·41 to 16·47]; p=0·039). There were no significant differences between study groups in adherence to social distancing (β 5·95 [−1·56 to 13·45]; p=0·12) or handwashing (β 5·11 [−1·59 to 11·80]; p=0·14).

Second, we used Markov Chain Monte Carlo imputation methods to impute missing outcome measures for those who completed at least one biweekly smartphone survey. Based on Rubin,^37^ we pooled analytic results from five multiple imputed datasets into a single result. Compared with the standard of care group, participants in the onsite point-of-care testing and education group had non-statistically significant higher masking behaviours (β 8·00 [95% CI −0·56 to 16·57]; p=0·07). Social distancing (β 4·98 [−2·77 to 12·74]; p=0·21) and handwashing (β 2·82 [−3·78 to 9·41]; p=0·40) were not significantly different between study arms.

Generalised estimating equations models examined the association between onsite point-of-care testing and education intervention dose and adherence to mitigation measures while accounting for individual-level correlations (table 2). For these analyses, we used only data from participants in the intervention group. Bivariate analyses showed that the intervention dose was significantly associated with subsequent adherence to masking (β 4·90 [95% CI 1·47 to 8·33]; p=0·0050). There were no statistically significant differences according to the intervention dose in social distancing (−3·22 [–7·68 to 1·23]; p=0·16) or handwashing (0·15 [−2·86 to 3·15]; p=0·92). Results based on multiple imputations were not materially different.

## Discussion

This study examined whether onsite SARS-CoV-2 testing and education provided by a trained community health worker at a re-entry-focused community-based organisation improved testing uptake and adherence to mitigation behaviours compared with a referral for offsite testing. We found that participants in the intervention group, compared with the standard of care group, had greater odds of completing at least one SARS-CoV-2 test and were tested more times over the 12-month period. Participants also reported greater adherence to masking behaviours than the standard of care group, with no substantial differences in results based on complete-case analysis versus multiple imputations for missing data. No significant differences were detected related to social distancing and handwashing. Among participants in the onsite point-of-care testing and education group, there was an association between intervention dose (the amount of contact with the community health worker) and adherence to masking but not adherence to social distancing and handwashing.

This trial extends the literature on community health worker or peer-delivered biomedical testing and counselling interventions for formerly incarcerated individuals. We chose to incorporate a community health worker with lived experience of incarceration into our intervention to address barriers, such as medical mistrust, discrimination, and stigma; problems that formerly incarcerated individuals face in accessing health-care services.^27^ Community health worker interventions, particularly those that address social–environmental factors during re-entry such as stigma, accompaniment to medical appointments, and transportation assistance, are well studied in HIV care^30^ and post-incarceration models in which trained community health workers build trust and provide support to improve engagement in health care.^28,29^ For this intervention, we trained the community health worker to use a point-of-care PCR platform for SARS-CoV-2 testing and deliver COVID-19 education. The intervention increased testing uptake, particularly for additional SARS-CoV-2 tests following the initial testing and education visit, and masking behaviours, which might have been due to the relationship built with the community health worker. Additional qualitative data that will be reported elsewhere assess the quality of participant and community health worker interactions. Expanded roles for community health workers beyond patient navigation could also be applied to testing and education regarding other respiratory pathogens such as influenza and tuberculosis, as well as other infectious diseases such as HIV, HCV, and sexually transmitted infections (STIs), in settings without access to other medical providers. The high proportion of participants in our sample who reported they first had COVID-19 while in jail or prison suggests the importance of community health worker-led interventions within carceral settings themselves.

Testing uptake might also have been affected by co-locating the intervention at a re-entry-focused community-based organisation. Other studies have demonstrated success with co-locating HIV testing services onsite at community-based programmes, such as drug treatment programmes,^36,38^ as well as co-locating HCV testing and buprenorphine treatment into harm reduction agencies.^39^ Co-location addresses structural factors by reducing waiting times, costs, and travel to traditional health-care sites, and for historically marginalised groups, might reduce health care-related stigma and mistrust. Although asymptomatic SARS-CoV-2 testing is no longer a recommended intervention, the study has important implications for delivering care during re-entry. In a US setting with access to Medicaid, assistance from a community-based organisation, and community testing services, referral was unsuccessful for approximately half of the participants in the standard of care group. Although we cannot be sure why onsite point-of-care testing and education led to greater testing uptake, we hypothesise it was related to convenience of co-location and the efforts of the community health worker. Community-based organisations could deliver testing for other pathogens such as HIV, HCV, and STIs after re-entry, and delivering integrated health services is a reliable strategy to increase utilisation. In a future pandemic, community-based organisations could deliver expanded testing and care for respiratory viral pathogens. Moreover, given that our study population reported high rates of living in congregate settings, this model could also play a role in risk mitigation in community-based congregate settings such as shelters.

The study has limitations. First, the lack of masking could have introduced bias; however, our protocol dictated equivalent participant outreach for each group, and completing SARS-CoV-2 testing was an objective outcome measure. Second, our participants were recruited from one community-based organisation in NYC, which could limit generalisability to organisations in suburban, rural, or other urban areas outside of NYC, as well as to formerly incarcerated individuals who are not accessing services at re-entry community-based organisations. Third, generalisability is affected by a single community health worker delivering the intervention as this might not be broadly representative of all community health workers and, therefore, we cannot make definitive statements of effects. Fourth, adherence to mitigation behaviours was self-reported, and we cannot objectively determine participants’ masking and other mitigation behaviours. Fifth, rates of loss to follow-up were suboptimal, reflecting a well known structural barrier among formerly incarcerated individuals and other marginalised populations. Further research is needed to meet people where they are and support this population to remain engaged in care. Sixth, the study statistician analysed interim results for Data and Safety Monitoring Board meetings, and because we did not have formal stopping rules, type I error might not have been adequately controlled for. Finally, the changing political and public health landscape (eg, testing mandates) might have affected testing uptake and mitigation behaviours during periods of higher SARS-CoV-2 transmission; however, these period effects were randomly distributed between the study groups and will be a focus of a future analysis. Despite these potential limitations, our study provides an example of how communicable diseases such as COVID-19 could be effectively mitigated among formerly incarcerated individuals and other vulnerable populations.

Few interventions targeted formerly incarcerated individuals during the COVID-19 pandemic, despite their high burden of illness, but our study demonstrates that re-entry-focused community-based organisations could have played a greater role in testing and risk mitigation. Our community health worker-delivered SARS-CoV-2 testing and education intervention at a re-entry-focused community-based organisation increased uptake of SARS-CoV-2 testing and masking behaviours among formerly incarcerated individuals. Although further, larger trials are required, our intervention could be a model for other respiratory pathogens such as influenza and tuberculosis, as well as other infectious diseases such as HIV, HCV, and STIs. The MOSAIC model could also be extended to community-based organisations that serve other historically marginalised populations, providing important guidance for the COVID-19 pandemic and future pandemics.

## Supplementary Material

1

2

## Figures and Tables

**Figure:**
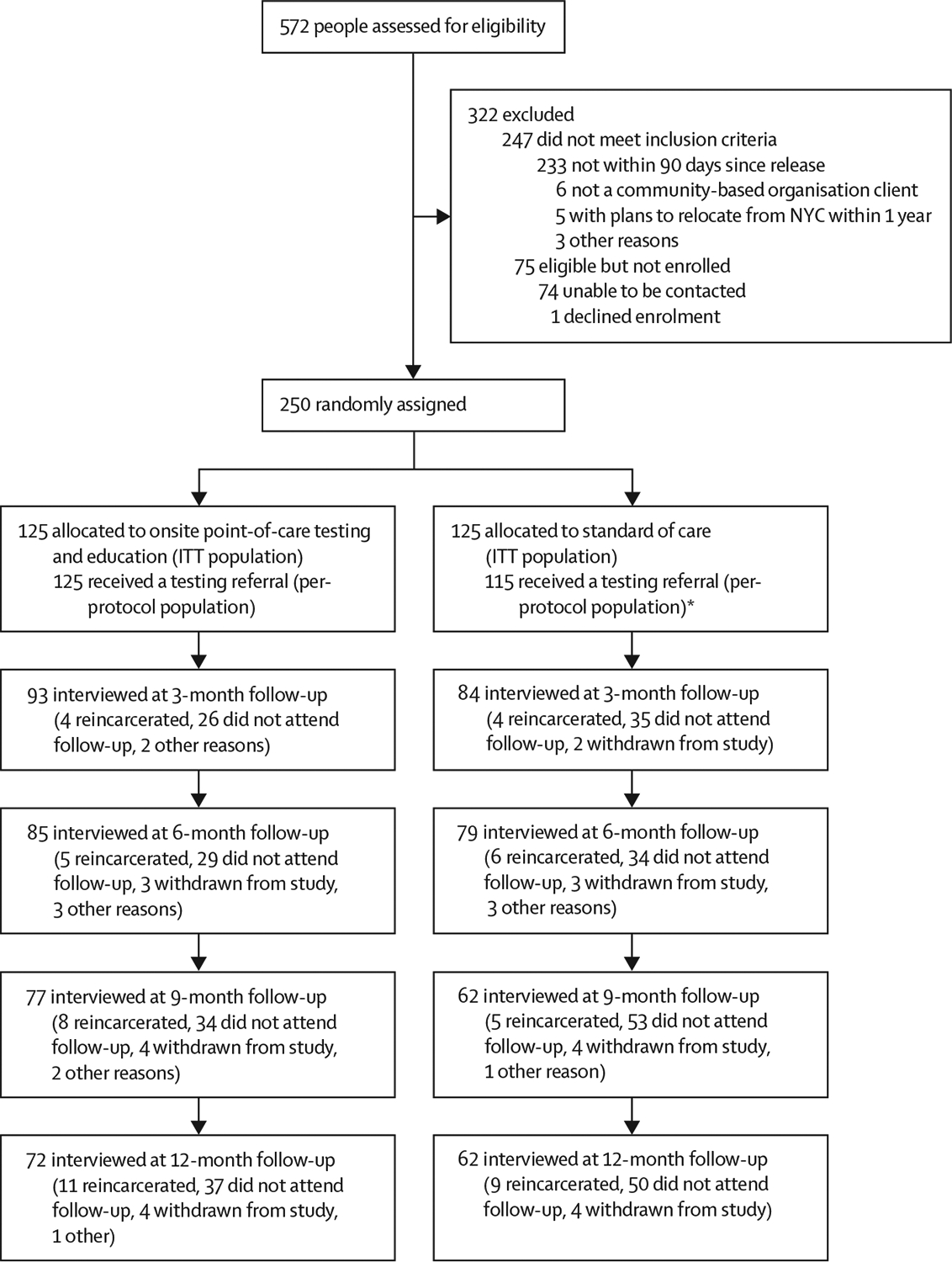
Trial profile The number of participants who were not included in the follow-up assessments at each timepoint are listed in each box in parentheses. Reincarcerated refers to an incarceration during the participant’s follow-up period. Did not attend follow-up refers to participants who either missed a visit due to scheduling challenges, did not show up to the appointment, or were lost to follow-up. Withdrawn from the study refers to participants who either opted to withdraw from the study or those who were withdrawn by the investigator. Other reasons includes less common reasons for not attending follow-up visits such as a participant unintentionally not being added to the follow-up calendar or death (n=1; unrelated to participation in the study). ITT=intention-to-treat. NYC=New York City. *Ten participants in the standard of care group did not receive a testing referral (nine were unable to be contacted and one had withdrawn from the study).

**Table 1:** Baseline characteristics

	Total (n=250)	Onsite point-of-care testing and education group (n=125)	Standard of care group (n=125)
**Sociodemographic characteristics**			
Age, years	42·0 (11·8)	42·8 (12·2)	41·2 (11·3)
Gender identity			
Cisgender men	216 (86%)	106 (85%)	110 (88%)
Cisgender women	30 (12%)	17 (14%)	13 (10%)
Transgender women	2 (1%)	2 (2%)	0
Non-binary or none of these describe me	2 (1%)	0	2 (2%)
Race and ethnicity			
Non-Hispanic Black	120 (48%)	69 (55%)	51 (41%)
Hispanic	82 (33%)	39 (31%)	43 (34%)
Non-Hispanic White	32 (13%)	12 (10%)	20 (16%)
Other	16 (6%)	5 (4%)	11 (9%)
Number of years incarcerated in lifetime	12·8 (11·6)	13·6 (11·7)	11·9 (11·4)
**Socioeconomic factors**			
Employment status			
Full time	11 (4%)	6 (5%)	5 (4%)
Part time	16 (6%)	6 (5%)	10 (8%)
Unemployed	155 (62%)	77 (62%)	78 (62%)
Highest level of education			
Did not complete high school	62 (25%)	33 (26%)	29 (23%)
General Educational Development or high-school diploma	104 (42%)	57 (46%)	47 (38%)
Some college	38 (15%)	13 (10%)	25 (20%)
College and higher	41 (16%)	19 (15%)	22 (18%)
Other	5 (2%)	3 (2%)	2 (2%)
Household income from legal sources			
<US$12 500	230 (92%)	113 (90%)	117 (94%)
≥US$12 500	16 (6%)	9 (7%)	7 (6%)
Missing	4 (2%)	3 (3%)	1 (1%)
Current housing status			
Transitional or permanent single-room occupancy*	70 (28%)	34 (27%)	36 (29%)
Shelter*	69 (28%)	35 (28%)	34 (27%)
Medical or behavioural supportive housing*	34 (14%)	19 (15%)	15 (12%)
Rent or own house or apartment	32 (13%)	17 (14%)	15 (12%)
Family or friends	40 (16%)	18 (14%)	22 (18%)
Other	5 (2%)	2 (2%)	3 (2%)
Number of days incarcerated in the past 30 days	3·2 (6·3)	2·7 (6·0)	3·6 (6·5)
Do not know or refused	7 (3%)	1 (1%)	6 (5%)
**Insurance and public assistance coverage**			
Insurance status			
Government health plan (eg, Medicaid, or Medicare)	208 (82%)	102 (82%)	106 (85%)
Private health insurance	2 (1%)	0	2 (2%)
Uninsured	40 (16%)	23 (18%)	17 (14%)
**Mental health conditions**			
Depression†	51 (20%)	23 (18%)	28 (22%)
Did not self-report depression	199 (80%)	102 (82%)	97 (78%)
Anxiety†	38 (15%)	14 (11%)	24 (19%)
Did not self-report anxiety	212 (85%)	111 (89%)	101 (81%)
Vaccine status			
None	58 (23%)	30 (24%)	28 (22%)
Incomplete (one dose)	9 (4%)	3 (2%)	6 (5%)
Fully vaccinated (two doses and no booster)	88 (35%)	43 (34%)	45 (36·%)
Fully vaccinated plus one booster	70 (28%)	37 (30%)	33 (26%)
Fully vaccinated plus two or more boosters	25 (10%)	12 (10%)	13 (10%)
Ever tested for COVID-19	239 (96%)	120 (96%)	119 (95%)
Do you think you have or ever had COVID-19?			
Yes, confirmed by positive test	85 (34%)	42 (34%)	43 (34%)
Yes, suspected by doctor but never tested	2 (1%)	2 (2%)	0
Yes, my own suspicion	39 (16%)	16 (13%)	23 (18%)
No	123 (49%)	65 (52%)	58 (46%)
Missing	1 (1%)	0	1 (1%)
Where were you when you first had COVID-19?			
Hospital, urgent care, or clinic	14/126 (11%)	5/60 (8%)	9/66 (14%)
Jail or prison	97/126 (77%)	47/60 (78%)	50/66 (76%)
Other	16/126 (13%)	8/60 (13%)	8/66 (12%)

Data are n (%), n/N (%), or mean (SD). Not all participants were comfortable reporting their income from legal sources.

* Merged as congregate settings in text.

† Participants self-reported data related to medical conditions.

**Table 2:** Primary and secondary analyses

	Effect estimate (95% CI)	p value
**Bivariate logistic regression model**		
At least one complete SARS-CoV-2 test during the 12-month study period	OR 5·9 (3·1 to 11·1)	<0·0001
**Bivariate Poisson regression model**		
Number of complete SARS-CoV-2 tests during the 12-month study (range=0–5)	IRR 2·4 (1·9 to 3·0)	<0·0001
**Bivariate GEE models for association of study group with adherence to mitigation behaviours**		
Masking	β 8·44 (0·41 to 16·47)	0·039
Social distancing	β 5·95 (–1·56 to 13·45)	0·12
Handwashing	β 5·11 (–1·59 to 11·80)	0·14
**Bivariate GEE models for association of intervention dose with adherence to mitigation behaviors**		
Masking	β 4·90 (1·47 to 8·33)	0·0050
Social Distancing	β −3·22 (–7·68 to 1·23)	0·16
Handwashing	β 0·15 (–2·86 to 3·15)	0·92

GEE=generalised estimating equation. IRR=incidence rate ratio. OR=odds ratio. β indicates the population-averaged effect, the average change in the outcome measure on a scale of 1–100.

## Data Availability

Individual de-identified participant data that underlie the results reported in this Article, as well as the study protocol, statistical analysis plan, and analytic code, will be available upon request beginning 9 months and ending 36 months following Article publication for researchers who provide a methodologically sound proposal, subject to approval by an independent review board and a signed data access agreement. Proposals should be directed to matthew.akiyama@einsteinmed.edu to gain access.

## Research in context

### Evidence before this study

We searched PubMed for studies published up to May 25, 2025, (the date our last participant completed the trial) using the following search string: “SARS-CoV-2”[MeSH Terms] OR “SARS-CoV-2”[tiab] OR “COVID-19”[MeSH Terms] OR “COVID-19”[tiab] OR COVID19[tiab]) AND (“Community Health Workers”[MeSH Terms] OR “community health worker”[tiab] OR CHW[tiab] OR peer[tiab] OR “peer navigator”[tiab] OR “community-based organization”[tiab]) AND (education[tiab] OR testing[tiab]) AND (randomized[tiab] OR “randomized controlled trial”[Publication Type]). No randomised studies examined testing or education strategies among incarcerated or previously incarcerated individuals. Four randomised controlled trials examined COVID-19 testing or mitigation interventions. In Mali, a community-based, community health worker-delivered, integrated COVID-19 and malaria testing strategy increased testing uptake compared with referral to nearby health facilities. In South Africa, among COVID-19 index cases identified at public clinics, a home-based, community health worker-delivered infection mitigation intervention (STOPCOV), which included provision of face masks, hand sanitiser, and written educational materials, was no more effective than standard of care. In the USA, among Native American and Latino adults, community health worker support increased COVID-19 home testing compared with standard of care. Finally, San Diego’s LinkUP intervention, which delivered tailored COVID-19 education, motivational interviewing, problem-solving, and planning by trained peer counsellors, significantly increased COVID-19 testing uptake among people who inject drugs compared with the control group. In this population of people who inject drugs, 12·7% had been incarcerated 6 months before the study and incarceration was associated with lower COVID-19 testing uptake in a univariate analysis.

### Added value of this study

Our study provides high-quality evidence that an onsite community health worker-led point-of-care COVID-19 testing and education intervention at a community-based organisation serving formerly incarcerated individuals led to increased COVID-19 testing and masking behaviours compared with standard referral to offsite testing. During the 12-month study period, participants in the intervention group were 5·9 times (95% CI 3·1–11·1; p<0·0001) as likely to have at least one complete SARS-CoV-2 test compared with standard referral. The incidence rate ratio of complete SARS-CoV-2 tests was 2·4 times (95% CI 1·9–3·0; p<0·0001) as high among participants in the onsite point-of-care testing and education intervention group versus standard of care. Participants in the intervention group had significantly higher masking behaviours compared with standard referral (β 8·44 [95% CI 0·41–16·47]; p=0·039). Social distancing and handwashing were not found to be statistically significant between the groups (β 5·95 [−1·56 to 13·45]; p=0·12) and (β 5·11 [−1·59 to 11·80]; p=0·14), respectively. Intervention dose (ie, the number of testing and education visits with the community health worker) was significantly associated with greater adherence to masking (β 4·90 [1·47–8·33]; p=0·0050), but not social distancing (β −3·22 [−7·68 to 1·23]; p=0·16) and handwashing (β 0·15 [−2·86 to 3·15]; p=0·92).

### Implications of all the available evidence

Community health workers can provide health system navigation and disseminate health-related information, and emerging evidence supports community health worker-delivery of point-of-care testing for infectious diseases. We did not find any interventions that specifically targeted community-dwelling previously incarcerated individuals during the COVID-19 pandemic. Our study shows how community health workers could play a crucial role in increasing infectious diseases testing and behavioural modification among formerly incarcerated individuals. Co-locating our intervention in a community-based organisation where previously incarcerated individuals access important wraparound services might have also played an important role. These findings have important implications for public health interventions to reduce the spread of respiratory viruses, such as SARS-CoV-2, but might also be relevant for other infectious diseases such as influenza, tuberculosis, HIV, and hepatitis C virus, among communities that face barriers to care.
