# Supplementary material for: Mitigation of COVID-19 through onsite testing and education among formerly incarcerated individuals (the MOSAIC study): an open-label, single-centre, randomised controlled trial
Source: Lancet Public Health. Author manuscript; Available in PMC 2026 Jul 7. (PMC13338770; doi:10.1016/S2468-2667(26)00093-9)
Supplement: 1 [file NIHMS2190773-supplement-1.pdf]

THE LANCET
Public Health

Supplementary appendix 1

This appendix formed part of the original submission and has been peer reviewed. We post it as supplied by the authors.

Supplement to: Akiyama MJ, Kaba-Diakite F, Dimaulaluan M, et al. Mitigation of COVID-19 through onsite testing and education among formerly incarcerated individuals (the MOSAIC study): an open-label, single-centre, randomised controlled trial. *Lancet Public Health* 2026; **11:** e457–67.

Mitigation Through On-Site Testing & Education Among Formerly Incarcerated Individuals Against Covid-19

"Leveraging community health workers to improve SARS-CoV-2 testing and mitigation among criminal justice-involved individuals accessing a corrections-focused community-based organization"

Principal Investigator: Matthew Akiyama, MD, MSc
Albert Einstein College of Medicine, Montefiore Medical Center
3300 Kossuth Ave, Bronx, NY 10467

Sponsor: National Institutes of Health
9000 Rockville Pike, Bethesda, Maryland 20892
Protocol ID: 2021-12976
Version 1.17 (March 27, 2024)

Table of Contents

Abbreviations 3
Abstract 4
Hypotheses 7
Objectives 7
Design and methodology 8
Data management 12
Ethical considerations 16
Data Safety and Monitoring Plan 21

Abbreviations

AE	Adverse event
CBO	Community-based organization
CDC	Centers for Disease Control and Prevention
CITI	Collaborative Institutional Training Initiative
CHW	Community Health Worker
CLI	Criminal legal-involved individuals
CLIA	Clinical Laboratory Improvement Amendments
COVID-19	coronavirus 2019 (COVID-19)
DHHS	U.S. Department of Health and Human Services
DOC	Department of corrections
DSMB	Data and Safety Monitoring Board
EMR	Electronic medical record
EUA	Emergency use authorization
FDA	Food and Drug Administration
FWA	Federal Wide Assurance
HCV	Hepatitis C Virus
HIPAA	Health Insurance Portability and Accountability Act
HIV	Human immunodeficiency virus
ICER	incremental cost effectiveness ratios (ICERs)
IRB	Institutional review board
NSP	Needle and syringe programs
OHRP	Office of Human Research Protection
O-PoC	Onsite-point-of-care
PPE	Personal protective equipment
QALY	Quality adjusted life year
RCT	Randomized control trial
SARS-CoV-2	Severe acute respiratory syndrome coronavirus 2
SAE	Serious adverse event
SCT	Social cognitive theory
SoC	Standard of Care
US	United States of America
YFC	Years of full capability

Abstract

The United States (U.S.) has experienced higher mortality than any other nation due to COVID-19 with nearly 13.5 million cases and over 268,103 deaths. Due to the limited ability to socially distance, poor ventilation, and limited hygiene supplies, U.S. prisons and jails have observed explosive transmission of SARS-CoV-2 accounting for the 10 largest U.S. outbreaks. Because 95% of criminal justice-involved individuals reenter society COVID-19 transmission extends beyond those who are currently incarcerated. As justice-involved individuals reenter the community, they face high rates of homelessness, and many others live in other congregate settings such as converted hotels and halfway houses. The increased risk of SARS-CoV-2 while incarcerated coupled with the likelihood of living in congregate settings after incarceration, create conditions ripe for rapid COVID-19 transmission that will be critical to address in order to gain control of COVID-19 in the U.S. The goal of this study is to test the impact and cost-effectiveness of an intervention to mitigate SARS-CoV-2 transmission among justice-involved individuals recently released from incarceration. We will conduct a randomized trial to compare the effectiveness of an onsite Point-of-Care (o-PoC) SARS-CoV-2 testing and education intervention with community health workers (CHWs) as a central component compared to the standard of care at a community-based organization (CBO) that provides services to justice-involved individuals in New York City. We will measure costs of testing, education, and navigation, and explore the cost-effectiveness of the onsite Point-of-Care intervention compared to the standard of care. Our specific aims are to: 1) Test the effectiveness of an onsite PoC SARS-CoV-2 intervention in a corrections-focused CBO; 2) Model the cost-effectiveness of an onsite PoC SARS-CoV-2 intervention among CLICLI compared to SoC. Because testing, education, and navigation will be provided by CHWs in a culturally-sensitive environment and test results will be received in minutes (rather than days), we hypothesize that O-PoC will be associated with improved testing uptake and receipt of test results, mitigation behaviors (mask wearing, hand hygiene, social distancing), and those who attend more O-PoC sessions will have better adherence to mitigation behaviors.

Background and justification for the study

The United States (U.S.) has experienced higher mortality than any other nation due to coronavirus 2019 (COVID-19) with nearly 13.5 million cases and over 268,103 deaths.[1] U.S. prisons and jails play an important role in the transmission of severe acute respiratory syndrome coronavirus 2 (SARS-CoV-2),[2,3] accounting for the 10 largest U.S. outbreaks.[4] Because 95% of criminal legal-involved individuals (CLICLI) reenter society, SARS-CoV-2 transmission extends beyond those who are currently incarcerated. For example, in the spring of 2020, CLI were estimated to account for 16% of all infections in Illinois.[5] As CLI reenter the community, they face high rates of homelessness – 50,000 CLI enter a homeless shelter each year,[6] and many live in other congregate settings. Increased risk of SARS-CoV-2 while incarcerated, coupled with the likelihood of living in congregate settings after incarceration,[7,8] create conditions ripe for SARS-CoV-2 transmission.

CLI are likely to be at high risk of SARS-CoV-2 infection and poor outcomes from COVID-19 due to overrepresentation of associated risk factors. Risk factors for SARS-CoV-2 infection include being a person of color, low education level, and low socioeconomic status.[9,10] Risk factors for severe presentations of COVID-19 include having immunocompromising conditions or other co-morbidities, all of which are more common in CLI compared to the general population.[11] In addition, barriers to care and medical mistrust among CLI could lead to undiagnosed infection or delays in treatment,[12-16] leading to poor individual outcomes as well as onward transmission. These devastating impacts coupled with overwhelming costs including a $16 trillion loss in the U.S. due to the pandemic-induced recession and the economic effects of shorter and less healthy lives[17] necessitate novel interventions to improve COVID-19 mitigation among vulnerable populations like CLI.

Correctional health is community health. This proposal focuses on CLI, a socially vulnerable population, in which SARS-CoV-2 infection rates as high as 96% have been observed.[18] This is of particular concern as SARS-CoV-2 screening procedures vary among correctional institutions, and in many cases only those with exposure risk factors and symptoms are tested.[19] Moreover, 50% or greater have asymptomatic infections in custodial settings.[20,21] An outbreak in a single jail can impact transmission for an entire region because persons scatter widely upon release. Recent data linked the cycling of 2,129 people through the Cook County Jail in March 2020 to an additional 4,575 cases in Illinois the following month, suggesting that almost 1 in 6 cases were linked to individuals released from Cook County Jail.[22] Similarly, a modelling study of the 20 largest jails in the U.S. projected that county jail systems could lead to a greater than 200% increase in COVID-19 deaths in some jurisdictions within a 6-month period without effective mitigation.[23] This trend could have an even greater impact if decarceration efforts in response to the COVID-19 pandemic are scaled up. Thus far, decarceration efforts have led jails and prisons to reduce their populations by 30% and 5%, respectively.[24]

SARS-CoV-2 testing in the U.S. has been inadequate, particularly among marginalized populations. The COVID-19 response in the U.S. has been plagued by delays in SARS-CoV-2 testing, long wait times, and slow turn around on test results.[25,26] While these parameters are improving for some populations, racial and ethnic minorities and individuals of lower socioeconomic status still face stark inequalities in access to testing.[27-30] Although data on testing rates in U.S. prisons and jails remain poor,[19] a lack of access to testing has been widely reported.[31,32] National recommendations include symptom-based and asymptomatic testing for contact tracing purposes, as well as those without any exposures for early identification (e.g. testing at intake and prior to transfer to another facility);[33] however, there are no recommendations regarding testing CLI as they return to the community, a process that may be rushed without adequate discharge planning, particularly during pandemic peaks.[34]

Asymptomatic community testing will be required to identify CLI at risk of transmission. Recent outbreaks of COVID-19 among individuals in correctional facilities and other congregate settings have demonstrated a large proportion of asymptomatic infections.[20,21,35] Additionally, anecdotal reports have emerged of CLI who were unaware of SARS-CoV-2 infection reentering the community and living in congregate settings post-release. For infections like HIV, exit testing strategies in correctional settings have rarely been adopted due to cost and low perceived benefit to correctional facilities;[36] therefore exit testing is unlikely to be implemented for COVID-19. Without increased access to community testing early after release, CLI and their contacts are at risk of community spread. As the number of incarcerated persons returning to the community grows, so will the need to provide appropriate services to this population. Testing is critical

for early intervention. Post-release, CLI often seek housing in congregate settings and transition from one setting with high rates of COVID-19 to another. Given this potential to infect those in the community they return to, it is important to ramp up accessible testing in high-risk communities.

Community-based organizations (CBOs) provide critical reentry services to people released from jail and prison. The nearly 400 corrections-focused CBOs located across the U.S. are a critical link to supportive services for CLI.[37] These services include housing, employment, substance use treatment, and healthcare. Corrections-focused CBOs often have staff who are onsite in jails and prisons or partner with correctional discharge planners to provide a 'warm hand off' so that the needs of their clients (often elicited through structured needs assessments)[38] are known upon reentry to the community. In addition to promoting continuity of care and coordinated re-entry, CBOs are trustworthy in comparison with healthcare institutions and providers.[14] Over $262 million in government funding bolsters re-entry services with another $100 million anticipated in 2021.[39] Leveraging CBOs to provide SARS-CoV-2 testing could serve as part of a national strategy to improve mitigation of COVID-19 among CLI.

An onsite point-of-care (PoC) intervention could be the missing link in improving COVID-19 mitigation for CLI. Traditional healthcare settings are often underutilized by marginalized populations, such as CLI, due to factors like medical mistrust and competing priorities such as homelessness and substance use disorders.[12-16] Similar to PoC HIV and HCV testing, SARS-CoV-2 PoC testing at CBOs among CLI and other marginalized populations is likely to increase uptake and be cost-effective.[40-42] Due to the rapidity with which SARS-CoV-2 spreads, if test results are not provided rapidly, they are of low utility in guiding quarantine, contact tracing, and social distancing. PoC SARS CoV-2 PCR testing provides reliable results (sensitivity, specificity: 100%)[43] in minutes rather than hours or days. For example, the Cepheid Xpert **Xpress** SARS-CoV-2 PCR test provides rapid detection of the current SARS-CoV-2 infection in less than 30 minutes with less than a minute of hands-on time to prepare the sample.[44] The Cepheid Xpert **Xpress** SARS-CoV-2 PCR test has received FDA emergency use authorization (EUA) approval and is Clinical Laboratory Improvement Amendments (CLIA) waived.[45] Therefore, the Cepheid Xpert **Xpress** SARS-CoV-2 PCR test is simple and appropriate for decentralized use in non-laboratory settings.

Community health workers (CHWs) with lived experience of incarceration provide culturally sensitive, cost-effective care. CHWs help to circumvent barriers CLI face, such as medical mistrust, discrimination, and stigma.[46] Innovative post-incarceration care coordination models with trained CHWs build trust with CLI and provide support, which improve linkage to healthcare services after incarceration.[47-49] CHW interventions are well studied in HIV care,[50-52] and are promising for CLI with other conditions. The Centers for Disease Control (CDC) and others have called for more interventions that leverage CHWs to provide COVID-19 related care.[53-57] CHWs can also conduct needs assessments for participants related to housing and access to masks and hygiene supplies, and facilitate access to these resources. However, to date, CHW interventions that reduce transmission and promote positive COVID-19 health outcomes among vulnerable populations have not been rigorously studied. Moreover, interventions that leverage CHWs to provide point-of-care testing for infectious diseases in high-income settings have been understudied. Thus far, CHWs have been only been leveraged to perform rapid diagnostic tests for diseases such as malaria or HIV in low-income countries.[58,59] CHWs tend to be less costly; therefore, employing CHWs to provide SARS-CoV-2 testing and education after incarceration may also be cost-effective.

Cost-effective strategies are needed to mitigate the COVID-19 pandemic. In the early days of the COVID-19 pandemic, decisions about interventions, including school closures, mask wearing, and testing options, were made on the basis of limited information. The cost-effectiveness of lockdowns and other initial interventions are not known. But, preliminary evidence suggests that swift contact tracing and isolation are likely to be highly cost-effective.[60] As the pandemic continues, and as COVID-19 transitions become endemic, a range of interventions including testing and vaccination should be explicitly evaluated for cost-effectiveness. Our proposed intervention, onsite point of care (O-PoC), which will facilitate early case identification and provide counseling to prevent transmission, is likely to be cost-effective in limiting transmission of COVID-19 compared with the standard of care (SOC).

Interventions are needed to improve uptake of mitigation measures among CLI. Behaviors that reduce the risk of SARS-CoV-2 transmission and are recommended by the CDC include: 1) mask wearing, 2) hand hygiene, and 3) social distancing.[61] Despite mixed evidence on the impact of masks for influenza

and other respiratory viruses,[62] masks are effective in preventing transmission of SARS-CoV-2.[63-65] Modeling data estimate that an increase in mask wearing from the current 50% to 95% could have prevented roughly 100,000 deaths in the last quarter of 2020.[66] Hand hygiene and social distancing are also effective mitigation strategies to reduce SARS-CoV-2 transmission.[67,68] For mitigation strategies, effectiveness of reducing transmission is dependent on adherence.[64] For example, in a randomized controlled trial (RCT) of influenza transmission, greater adherence led to lower transmission risk for mask wearers.[69] Uptake of mitigation measures among marginalized communities is concerning: A U.S. national survey study to measure differences in knowledge, beliefs, and behavior about COVID-19 suggested Black participants, men, and people younger than 55 years had less knowledge than other groups, and Black participants were more likely than Whites to report being infected or know someone who tested SARS-CoV-2 positive. Yet, knowledge of COVID-19 symptoms and preventive behaviors was lower among Black and Latinx respondents, and people younger than 30 years than Whites.[70] The demographic factors in this national survey are largely reflective of U.S. criminal justice system. Uptake of these measures among CLI may be even more limited due medical mistrust, reduced access to masks and hand hygiene, and ability to socially distance due to living in congregate settings during and after incarceration.

Social Cognitive Theory guides our intervention. We seek to understand why CLI accept SARS-CoV-2 testing and adhere to mitigation behaviors. According to Social Cognitive Theory (SCT), several key determinants affect behavior,[71] and these determinants guide our intervention and evaluation. To change behaviors, *knowledge* is a precondition, and one must believe that s/he can control his/her health (*self-efficacy*). Change then occurs when one: 1) recognizes the *expected outcomes* (harms or benefits) that may result from his/her behaviors, 2) overcomes logistical *barriers and facilitators*, and 3) sets *goals*, with a strategy to achieve them. SCT also posits that learning occurs in a social context with a dynamic and reciprocal interaction of the person, environment, and behavior. Due to SCT's emphasis on social influence, and external and internal social reinforcement, it is an apt theoretical model for our CHW-led intervention that will occur onsite in a corrections-focused CBO. Using SCT to guide our understanding of the challenges (and potential solutions) associated with SARS-CoV-2 testing and adherence to mitigation measures post-incarceration, our O-PoC intervention will address: 1) *COVID-19-related knowledge*, 2) *barriers to and facilitators of SARS-CoV-2 testing and adherence to mitigation measures*, 3) the *expected outcomes from SARS-CoV-2 testing and mitigation measures*, and 4) *goals for SARS-CoV-2 testing and adherence to mitigation measures and strategies for reaching them*.

Summary and public health impact: Given the likelihood of COVID-19 remaining an endemic disease among high-risk populations, establishing effective mitigation interventions will be critical to stemming community transmission. A novel SARS-CoV-2 screening strategy that leverages CHWs to deliver SARS-CoV-2 testing, education, and navigation to sites that maximize socially distancing and vaccine uptake for CLI in corrections-focused CBOs will be a critical step in stemming community transmission among this high-risk, vulnerable population. Given there are nearly 400 corrections-focused CBOs in the U.S.,[72] onsite testing will be generalizable to CLI across the U.S. and can inform onsite testing strategies for other high-risk populations such as clients of homeless shelters or syringe exchange programs, and people living with other conditions, such as HIV or hepatitis C virus (HCV) infection.

Hypotheses

1. O-PoC (vs. SoC) will result in a greater proportion of tests performed and results received.
2. O-PoC (vs. SoC) will be associated with increased mitigation behaviors.
3. Higher O-PoC intervention dose will be associated with better adherence to mitigation behaviors.
4. O-PoC (vs. SoC) will be cost-effective in terms of cost per COVID-19 case identified and averted.

Objectives

1. Test the effectiveness of an onsite PoC SARS-CoV-2 intervention in a corrections-focused CBO.

2. Model the cost-effectiveness of an onsite PoC SARS-CoV-2 intervention among CLI compared to SoC.

Design and methodology

A. Overview

We will conduct a randomized trial to assess the effectiveness of an onsite Point-of-Care testing and education intervention (O-PoC) compared to standard of care (SoC) at the largest CBO providing services to CLI in New York City, The Fortune Society (Fortune). The current SoC includes a referral to offsite community testing. Guided by Social Cognitive Theory, in O-PoC we will employ CHWs who have lived experience of incarceration to provide: 1) COVID-19 education; 2) SARS-CoV-2 testing with Cepheid Xpert Xpress PCR tests at Fortune facilities; 3) Needs assessments and facilitated access to masks and hygiene supplies; 4) Navigation to vaccination sites (when available) and single-room housing at Fortune's supportive housing sites and partnering shelters, or alternative strategies that will maximize the ability to socially distance for those who test PCR positive; 5) Supportive counseling.

We will randomize 250 CLI to O-PoC or SoC. Over 12 months, participants in both groups will receive education about the importance of asymptomatic testing and be advised to test every 3 months. Data sources will include 5 questionnaires administered by study staff (0, 3, 6, 9, 12 months) and 26 web-based surveys via the Ethica application pre-loaded on the study smartphones (every 2 weeks over 12 months), Fortune program logs, and healthcare records. Because testing, education, and navigation will be provided by CHWs in a culturally-sensitive environment and test results will be received in minutes (rather than days), we hypothesize that O-PoC will be associated with improved testing uptake and receipt of results (primary outcomes), mitigation behaviors (mask wearing, hand hygiene, social distancing) (secondary outcomes), and those who attend more O-PoC sessions will have better adherence to mitigation behaviors. We will measure costs of testing, education, and navigation. For those who testing positive, we will assess the change in number and type of contacts to understand the risk of onward transmission mitigated by O-PoC compared with SoC. We will use a Markov model to evaluate the cost and impact of O-PoC among participants and their immediate contacts. We will explore cost-effectiveness of O-PoC compared to SoC in terms of cost per case identified and secondary cases averted. In sensitivity analysis, we will also explore the impact of high vs. low prevalence conditions and high vs. low sensitivity tests (e.g. PoC PCR vs. antigen) on cost and cost-effectiveness of O-PoC.

B. Study sites

We will recruit 250 CLI accessing services at Fortune a CBO providing reentry services to CLI in New York City, and which currently refers clients to offsite community testing for SARS-CoV-2.

<u>Fortune Society</u>

The Fortune Society is a CBO whose mission is to support successful reentry from incarceration and promote alternatives-to-incarceration (ATI). Fortune serves roughly 7,000 justice-involved individuals per year throughout New York City. Their clientele is mainly comprised of males (87%) and is predominantly within the age groups of 25-34 (28.3%) and 35-50 (31.7%). The majority are also unemployed (85.5%) at intake. Fortune has secured funding from New York City Health + Hospitals Test & Trace Community Based Organization Engagement Opportunity. As a result, the CBO is partnered with NYC Health + Hospitals to contribute to community safety, education, and awareness efforts around COVID-19. Fortune's administrative and primary service site is located in Long Island City, NY. They also have two residential housing facilities in Manhattan - Castle Gardens which is located in West Harlem and offers 62 beds in single and shared occupancy units for formerly incarcerated individuals who are homeless, and Freedom House in East Harlem which provides housing to 38 formerly incarcerated individuals. Fortune main location in Long Island City provides a range of services to individuals related to re-entry including facilitating access to education, employment, affordable housing, substance abuse treatment, healthcare, and family services to

build a strong foundation in their transition back to the community. Additionally, Fortune developed a team of CHWs to provide COVID-19 outreach efforts to Fortune's clients, including demystifying the contact tracing process, educating clients on personal protective equipment (PPE) as use, social distancing, getting tested, and, if positive, the quarantining process. CHWs also receive HIPAA/Privacy/Confidentiality training at onboarding as well as annually.

C. Study population and Recruitment

All subjects will be recruited from Fortune Society's existing community dwelling clientele, which is representative of the NYC jail population. The NYC jail population is comprised of mostly minority groups (49.5% Black, 24% Hispanic); therefore, we expect to have no trouble recruiting minority groups. We expect similar proportions in our study subjects. Although 90% of inmates in the NYC jail system and Fortune clients are male, we will attempt to balance the ratio of male to female participants as closely to 1:1 (50% male and 50% female) as possible to ensure our sample is as representative as possible.

**Inclusion criteria**	**Exclusion criteria**
1) Fortune Society clients<br>2) Greater than or equal to 18 years old.<br>3) Fluent in English or Spanish.<br>4) Resident of NYC<br>5) Released from a jail or prison system within 90 days.	1) Inability to provide informed consent.<br>2) Inability to complete study visits over 12 months.<br>3) Does not plan to reside in the NYC area for the next year.<br>4) Terminal illness

Individuals on parole are eligible to enroll in and participate in the study. If a participant is reincarcerated after being enrolled, they will remain as part of the study throughout the study period. During the re-incarceration period, the research team will not be in contact with the participant nor will any research-related activities be conducted until they are released back into the community. As a result, these individuals will not complete the REDCap interviews nor the web-based questionnaires during the weeks they are incarcerated.

Recruitment

We will utilize Fortune existing reentry service to provide active referral to the study for clients who meet eligibility criteria as outlined above. Case workers at Fortune will inform potential participants about the study during telephone check-ins and visits. Passive recruitment will occur by posting study flyers at Fortune Society locations. Eligible and interested patients will meet with the study coordinator (SC) over the phone or in-person for screening to determine eligibility. If eligible, they will complete informed consent for enrollment. Informed consent will be obtained in the patient's native language – English or Spanish. If necessary, an interpreter will be used, and the informed consent form will be written in that language. Other collaborating sites include the University of Bristol. No potential participants will be recruited or followed at this site.

D. Procedures

All study-specific procedures will be performed by research staff members (study physicians, nurses and research assistants), although some data collected in the study will be through routine clinical care. All study procedures will be performed with medical supervision. In addition to interviews, the primary study procedures involve nasal sample collection. Research staff have direct experience working with this population.

a. Questionnaires

Data will be collected using REDCap questionnaires at each research visit (0, 3, 6, 9 and 12 months). Research visits for both arms will occur in private rooms at Fortune

or remotely over the phone/videochat to maintain social distancing, the REDCap will be administered by the SC at each visit. The REDCap interviews and questionnaires will collect key socio-demographic factors, comorbidities, COVID-19-related knowledge. These interviews will also be used to ascertain the barriers to testing including attitudes toward COVID-19, mistrust of the healthcare system, and perceptions of risk as well as self-reported behavioral changes (e.g. mask wearing), ability to socially distance, and take other protective measures in the community. REDCap interview data will be directly entered on a laptop computer (by the SC if collected over the phone or directly by the participant if collected in person). The study laptop will be password protected and will contain only participant study ID (no identifying information).

Participants will also complete 26 brief (2-5 minute) web-based questionnaires every two weeks over the study period. For each web-based questionnaire (every 2 weeks), participants will receive personalized links to a web-based questionnaire that will be accessible for 24 hours. Participants will choose how (text or email) and when (day of the week and time) to receive automated links. They will receive two automated reminders if questionnaires are not completed within 2 and 3 hours of the original message. If questionnaires are not completed within 12 hours, research staff will be automatically notified and will call participants. Web-based questionnaires will focus on mitigation measures (e.g. mask wearing, hygiene) and housing.

To be respectful of participants' responsibilities outside of the study, we will implement a visit window period. This means that while participants will be expected to complete their follow up visits 3 months after their previous study visit, they will be able to complete the visit within the following timeframe: one month prior and one month after their expected visit date. Please see the table below:

Overview of data collection

Aim, hypothesis	Construct	Research visit	Source
**Outcome measures**			
Aim 1, H1	Tests performed	0, 3, 6, 9, 12	Program Coordinator logs, healthcare records
Aim 1, H1	Results received, Time to receipt (days)	0, 3, 6, 9, 12	RC
Aim 1, H2	Mitigation measures	0, 3, 6, 9, 12<br>Every 2 weeks	RC<br>WQ
Aim 1, H3	Impact of intervention dose on mitigation	0, 3, 6, 9, 12<br>Every 2 weeks	RC<br>WQ
Aim 2, H4	Cost and resource use	0, 3, 6, 9, 12<br>Every 2 weeks	RC, WQ, Logs, healthcare records, observation
**Other covariate measures**			
All	Sociodemographics	0,	RC
All	Knowledge and attitudes toward COVID-19	0, 3, 6, 9, 12	RC
All	COVID-19 symptoms	0, 3, 6, 9, 12	RC, WQ
All	COVID-19 testing experience (modified*)	0, 3, 6, 9, 12	RC, WQ
All	Comorbidities and care engagement	0, 3, 6, 9, 12	RC
All	Mental health impacts of COVID-19, PHQ-9,^ GAD-7^, Coronavirus Anxiety Scale, Effects of COVID-19 Outbreak	0, 3, 6, 9, 12	RC
All	MOS Social Support	0, 3, 6, 9, 12	RC
All	Household impact of COVID	0, 3, 6, 9, 12	RC
All	Social distancing experiences	0, 3, 6, 9, 12<br>Every 2 weeks	RC, WQ
All	Housing situation	0, 3, 6, 9, 12	RC, WQ
All	Substance use behavior, ASI^	0, 3, 6, 9, 12	RC
All	Mitigation (hand hygiene, mask wearing)	0, 3, 6, 9, 12<br>Every 2 weeks	RC, WQ
All	Quality of life^	0, 3, 6, 9, 12	RC
All	Crime and legal activities^	0, 3, 6, 9, 12	RC
All	Non-study Resource Utilization^	0, 3, 6, 9, 12	RC
All	Trust in the medical system and providers^	0, 3, 6, 9, 12	RC
All	Vaccine acceptability,^ and uptake	0, 3, 6, 9, 12	RC

RC=REDCap, WQ=Web-based Questionnaire. Measures in this study were selected from the PhenX Toolkit version August 27, 2020, Ver 33.5. except as otherwise noted (^). *Modified to capture SARS-CoV-2 testing experience onsite vs. offsite.

b. Records

Department of corrections records

Study Activity	Week	Timeframe
BL	0	Immediately after enrollment
Month 3	12	Opens on Week 8<br>Close on Week 16
Month 6	24	Opens on Week 20<br>Close on Week 28
Month 9	36	Opens on Week 32<br>Close on Week 40
Month 12	48	Opens on Week 44<br>Close on Week 52

We will utilize the DOC inmate lookup system. This system will be used to verify that potential participants were released from a jail or prison within the last 90 days. Date of release will also be confirmed by CHS partners. This system will also be used to assist with participant tracking. If the research team is unable to contact the participant through the contacts on their locator form, they will check the participant's re-incarceration status via the public DOC inmate lookup system.

Fortune program coordinator logs

We will use the Fortune Program Coordinator logs to determine testing uptake and the proportion of tests performed. The Fortune Program Coordinator will keep logs of: 1) all outreach calls made to clients to provide education about the importance of asymptomatic testing, 2) referrals to offsite testing (SoC arm), 3) tests performed, test results, and whether or not they were received by the client (O-PoC arm). The Program Coordinator will be trained by the Albert Einstein Collect of Medicine/Montefiore Medical Center team on obtaining and managing data for the logs.

As part of this study, we may also provide updated contact information or other needs the participant expresses to the research team to Fortune Society in an attempt to enhance the services they receive.

c. Specimen collection and laboratory procedures

Specimens

The specimens to be collected from enrolled subjects include nasal samples. Those randomized to receive offsite SARS-CoV-2 screening will have their tests collected by trained health professionals at their selected sites.

For individuals assigned to onsite testing, the PCR rapid analysis will be conducted by a CHW using the Cepheid Xpert® Xpress SARS-CoV-2 PCR Diagnostic Panel. Individuals randomized to onsite SARS-CoV-2 screening will self-swab for a nasal specimen under the supervision of the CHW at a time scheduled by the Fortune Program Coordinator (PC). CHWs will be trained in nasal collection by Cepheid and will oversee all onsite study-specific nasal collections. In the event a participant prefers not to self-swab, the CHW will collect the nasal sample. All specimens collected by the CHW will be identified by only a study identification number and no personal identifiers. Only selected research staff will have access to the codebook linking the subjects' study identification number.

For the onsite PoC group, the Einstein/Montefiore Project Coordinator will perform quality assurance every week for the first month and every 4 weeks thereafter to ensure test protocols are properly followed. Despite initial training and follow-up, there is a chance that the RT-PCR is administered incorrectly. However, SARS-CoV-2 tests will not be used to make decision about medical care, therefore results will not have serious health impacts if performed incorrectly.

d. Biosafety management

Authentication of Key Biological and/or Chemical Resources

Key biological resources in this proposal include nasal samples from human individuals. These are collected via nasal samples under the supervision of our Community Health Workers (CHWs) at Fortune Society. Samples will be collected and tested onsite at Fortune Society facilities where Cepheid Xpert Xpress SARS-CoV-2 PCR tests will be used to provide onsite point of care SARS-CoV-2 testing. Both sensitivity and specificity for the Xpert HCV Viral Load assay for HCV RNA quantification has been found to be 100%. It is our plan to introduce onsite Cepheid Xpert Xpress SARS-CoV-2 PCR to Fortune Society in coordination with this proposal.

Biohazards
Our study team has extensive experience working with infectious organisms, specifically with SARS-CoV-2, hepatitis c virus and HIV. We will ensure that any infectious or potentially infectious materials will be handled in appropriate containment facilities by fully trained and competent staff in accordance with national guidelines. CHWs will be trained in proper nasal collection and testing by Cepheid at Fortune Society and the Einstein/Montefiore Project Coordinator will perform quality assurance every week for the first month and every 4 weeks will to further ensure proper testing protocol. The Cepheid Xpert Xpress SARS-CoV-2 PCR will be administered by CHWs under a CLIA waiver. While there is a chance that the RT-PCR is administered incorrectly despite initial training and follow-up, since we will not utilize the SARS-CoV-2 tests results to make decisions surrounding medical care, we do not anticipate that an incorrectly administered test will cause serious health impacts.

Data management

A. Data storage

All study-specific procedures will be performed by research staff members (study physicians, nurses and research assistants), although some data collected in the study will be through routine clinical care. All study procedures will be performed with medical supervision. In addition to interviews, the primary study procedures involve nasal specimen collection. Research staff have direct experience working with this population and with HIV- and HCV-related research studies.

B. Data management

Prior to study enrollment, all research staff will be trained in principles of human subjects protection and maintenance of confidentiality of study participants. All staff will be required to complete CITI human subjects research training. In addition to the nasal sample collection training, CHWs will have already received HIPAA/Privacy/Confidentiality training from Fortune at onboarding as well as an annual refresher training.

To protect patient confidentiality, each participant will, upon enrollment, be assigned a unique identification number, which will be written on all data collection forms and specimens so that all results can be linked to the database, where the participant is only identified by this unique identification number. The participant's name will not appear on any data collection forms or in the study database.

All data collection instruments will be stored in closed, locked file cabinets with restricted access (i.e., in a locked room with only access by study personnel) at each study site. Consent forms and all participant identifying information obtained at study enrollment and used to track participants will be stored separately from data collection instruments, in separate locked filing cabinets. The electronic data will be stored in a password-protected web-based database that is compliant with the U.S. Health Information Portability and Accountability Act (HIPAA) requirements and is only accessible by approved study staff. The purpose of HIPAA regulations is to ensure that participants' personal health information is safe and secured from individuals or entities that are not authorized to use such information.

C. Data security

All paper forms will be kept in a locked file cabinet in a locked room. The database is located on a secure, encrypted web-based server, and all entries are logged with time stamps and user credentials. The databases also have a password expiry rule where one is asked to change their passwords periodically and are server authenticated. Study documents, including informed consent forms, source documents, and data collection forms, are stored at the study sites in a secure storage system. Storage areas are maintained securely and confidentially, filing cabinets will be locked and the doors to rooms housing the filing cabinets will be locked. Entry into record storage areas is restricted only to authorized staff.

D. Data monitoring and quality assurance

Data collected will be monitored by the Einstein Study Coordinator (SC), Fortune PC and Principal and Co-Investigators. The SC will review a subset of data collection forms and database entries for completeness and accuracy on a monthly basis from each site. The SC, and Dr. Akiyama will communicate on a weekly basis regarding enrollment and retention, study staff performance, database entry and cleaning.
On a monthly basis, the Einstein/Montefiore SC will perform quality assurance to ensure test protocols are properly followed as well as to monitor the following:

- Verify compliance with human subjects protections and other research regulations and guidelines, including confidentiality procedures, informed consent process, and regulatory documentation.
- Observe the performance of study procedures
- Assess adherence to the study protocol
- Confirm the quality and accuracy of information collected at the study site and entered into the study database
- Assess the resolution of any past or ongoing issues identified at previous monitoring visits

E. Data analysis

Analyses of data have been planned for each specific objective.

1) Test the effectiveness of an onsite PoC SARS-CoV-2 intervention in a corrections-focused CBO.
H1: O-PoC (vs. SoC) will result in a greater proportion of tests performed and results received. To test this hypothesis we will conduct separate logistic regression models with performing at least one SARS-CoV-2 test and receiving at least one SARS-CoV-2 test result during the 12-month study period as the dependent variables and study arm as the main independent variable. First, we will use bivariate logistic regression models to determine the effectiveness, i.e., the odds of performing a SARS-CoV-2 test and receiving a SARS-CoV-2 test results between randomization arms, O-PoC vs. SoC. Second, we will include key baseline variables (e.g., sex, race/ethnicity, history of SARS-CoV-2 testing and infections) that are unbalanced in the two groups in the multivariate analyses. In addition, we will also conduct separate bivariate and multivariate Poisson regression models to estimate the number of SARS-CoV-2 tests performed and the number of SARS-CoV-2 test results received during the 12-month study period with study arm as the main independent variable.
Power and sample size consideration: For H1, we have chosen to power on test results received because this is a more clinically meaningful in that tests that are received can produce actionable results. While there are no existing pilot data on SARS-CoV-2 testing to guide this power calculation, data comparing HIV and HCV test results received through onsite, point-of-care testing in settings serving other vulnerable populations such people who use drugs in a substance use treatment programs vs. offsite for those referred for laboratory-based HIV and HCV testing are ~70% and ~20%, respectively.[73,74] With 250 as our target sample size (125 per arm, assuming a 1:1 ratio), conservatively accounting for a 15% attrition, we will have a sample size of 212 (106 per arm) for H1. Table 1 shows this sample size will allow us to detect a tab difference in proportion of test results received (70% vs. 51%) with

Table 1. Minimum total sample size required to detect difference in proportion of tests results received between O-PoC and SoC

Power	O-PoC	SoC	Total N required
0.80	70%	51%	206
0.90	70%	48%	206
0.99	70%	41%	206
Assumptions: 1. two-tailed (alpha=0.05); 2. A 1:1 randomization ratio			

at least 80% of power at a significant level of 5% (two-sided test). To test the larger difference i.e. 70% vs. 20%, the power will be >99%.

H2: O-PoC (vs. SoC) will be associated with increased mitigation behaviors. Participants' 26 assessments of mitigation measures (e.g., mask wearing, hand hygiene, social distancing) for each 2-week time period will be the unit of analysis. We will determine the association between O-PoC testing (vs. SoC) and mitigation measures using mixed effect linear regression models (SAS Proc Mixed) that include individual specific random intercepts to account for hierarchical data (repeated measures within individuals). The primary dependent variable will be mitigation measures, such as frequencies of mask wearing hand hygiene, and social distancing (see section D.9.). The main independent variable will be randomization arm (O-PoC or SoC). The estimated betas will represent the effect in mitigation measures comparing O-PoC to SoC. We will also include key baseline variables (e.g., sex, race/ethnicity, history of SARS-CoV-2 testing and infections) that are unbalanced in the two groups in the multivariate analyses.

Table 2. Power obtained to detect difference in mitigation measures over time between onsite vs. offsite with N=106 per arm.

ICC	Cohen's d	Power
0.4	0.3	0.92
0.4	0.5	>0.99
0.6	0.3	0.80
0.6	0.5	>0.99
Assumptions: 1. two-tailed (alpha=0.05); 2. A 1:1 randomization ratio		

Power and sample size consideration (H2): We calculated power using simulated data (see Table 2). Since there are no available data from randomized studies that can guide our estimation of the effect size, we present several estimates with varying ranges of effect sizes (Cohen's d) and inter-class correlations (ICCs). In addition, our sample size estimates incorporate a two-tailed alpha level of 0.05. Table 2 shows that a sample size of 106 per arm (assuming a 1:1 ratio and a 15% attrition) can detect a moderate difference of 0.3 (Cohen's d) in mitigation measures 80% of the time with a more stringent condition (i.e., ICC=0.6). When ICC is smaller and the effect size is larger, the power will be greater.

H3: Higher O-PoC intervention dose will be associated with better adherence to mitigation behaviors. To test H3, we will examine data from O-PoC participants only. We will conduct mixed effect linear regression model to examine the association between O-PoC intervention dose and subsequent adherence to mitigation measures while accounting for individual level random effect. The independent variables will be attendance statuses at each O-PoC visit (0, 3, 6, 9 months), which is a time varying dichotomous variable. The dependent variable will be the corresponding 3-month summary scores (mean or proportion) of adherence to mitigation measures between visits. In addition, we will also conduct linear regression analyses to examine the association between the proportion of O-PoC attendance and overall mitigation measures over 12 months.

Power and sample size consideration (H3). Assuming the ratio of attendance to non-attendance is 7:3, with 106 O-PoC participants (assuming a 15% attrition) and 4 time points per individual, we will have 90% power to detect a moderate difference of 0.3 (Cohen's d) in mitigation measures between participants who will attend O-PoC visits vs. those who do not when ICC=0.4 and two-tailed alpha=0.05. Since O-PoC attendance is a time varying variable, the power will be greater when ICC is greater.

2) Model the cost-effectiveness of an onsite PoC SARS-CoV-2 intervention among CLI compared to SoC.

H4: O-PoC (vs. SoC) will be cost-effective in terms of cost per COVID-19 case identified and averted. We have argued that the O-PoC intervention will increase SARS-CoV-2 testing uptake and result provision and increase mitigation behaviors among CLI. We further hypothesize it will be cost-effective as we anticipate it will allow for early case identification and subsequent isolation and/or behavior change among those who are infected. Participants that are promptly informed of their status, receive education, will be more effectively able to isolate (reduce contact with others), more likely to perform additional mitigation measures such as mask wearing, hand hygiene, and social distancing, which will consequently prevent spread to others.

Methods for costing. O-PoC testing will involves set-up costs and intervention delivery costs, including materials and staff and CHW time for onsite testing, education, counseling, and patient navigation, while SoC involves the cost of referral, and implementation of SoC tests. Research associated costs will be excluded. Dr. Walker will consult with the Montefiore/Einstein Project Coordinator and Fortune staff to breakdown the intervention into cost centers/activities and to evaluate the ingredients that make up each activity. Resource use per individual will be recorded in study data (such as visits attended, supplies distributed, etc.), while the

average length of study visits will be estimated through operational questionnaires completed by Fortune staff on a regular basis (to achieve a random sample of visit/activity durations during each of the 5 intervention visits). Unit costs of consumables per activity or per month will be gathered from study and CBO expenditure records. The cost and resource use of COVID-19 treatment/hospitalization will be gathered from medical records and through reimbursement records. The bulk of the costing will be conducted during a two week visit by Dr. Walker to the intervention in year 4, during which activities will be observed, interviews conducted with study staff, and unit cost data gathered. The total and per study participant difference in cost between the OPoC intervention and offsite testing will be compared to the difference between cases identified in the two arms to calculate the incremental cost per case identified of O-PoC intervention vs. SoC.

Cost-effectiveness analysis. After measuring costs of O-PoC (vs. SoC) including staff time for testing, outreach and counselling, start up and overhead costs, and consumable costs, we will model the cost-effectiveness of O-PoC (vs. SoC) in terms of cost per case identified, cost per direct transmission event averted, and cost per quality adjusted life year (QALY) or years of full capability (YFC). In the cost-effectiveness analysis we will compare SARS-CoV-2 testing uptake between participants randomized to O-PoC vs. SoC. We will use a mathematical simulation model (Markov model) to evaluate for the cost and impact of testing within the cohort and their immediate contacts, this will assume a potential for transmission of COVID-19 from infected cases to close contacts (secondary attack rate) as reported in the literature through contact tracing and observational studies,[75,76] but will not account for the dynamics of further onward transmission due to uncertainty about how the initial epidemic will transition into seasonal outbreaks or how it will be affected by vaccination in the general population. As well as costs of testing, we will account for ED visits, hospitalization costs, and other healthcare required for COVID-19 cases. Healthcare benefits of the intervention will be evaluated using the EQ-5D and ICECAP-A questionnaires conducted at research visits, and converted into QALY and YFC, and incremental cost effectiveness ratios (ICERs) used to compare cost per QALY or YFC gained within intervention. This will account primarily for the improvement in quality of life achieved through engagement with healthcare, knowledge of COVID-19 status, and counselling received through the program. We will also incorporate projections of QALYs saved per infection averted, based on life table methods, adjusted for the age of the population and comorbidities.[77]

Sensitivity analyses. A sensitivity analysis will be performed using a range of values for testing uptake, social distancing measures, and population flow (number of O-PoC attendees; median/mean time to testing and follow-up), to evaluate the impact of varying our assumptions for key parameters on the cost-effectiveness. We will also model the way in which this strategy performs in high, moderate, and low prevalence settings, and using higher vs. lower sensitivity tests (e.g. PCR vs. antigen vs. saliva). The effect of false positives and false negatives will be incorporated into the model by assuming a proportion of those who test negative are false negatives and a proportion of those who test positive are false positives. For example, a systematic review revealed when comparing rapid PCR to antigen tests, the former had higher average sensitivity, 95.2% (95% CI: 86.7% to 98.3%).[78] Moreover, antigen tests had much greater variation in sensitivity when compared with that of rapid molecular assays. Recently, the FDA has issued an EUA for saliva-based laboratory diagnostic testing for SARS-CoV-2. Although analyses have identified specificity in saliva-based tests to be comparable other SARS-CoV-2 diagnostic measures, the newer test has a lower sensitivity than that of the nasopharyngeal PCR.[79] Therefore, while the quicker, easier and likely cheaper option for PCR collection than the standard nasal swab also eliminates the need for preservatives or specialized tubes for collection, current findings suggest that this may not be the most suitable option in settings where nasal PCR can be efficiently conducted.[37] The sensitivity analysis will help determine scalability and cost-effectiveness of SARS-CoV-2 testing strategies in other settings.

F. Qualitative data collection

We will conduct interviews with representative sub-set of 20-40 participants will be interviewed, all belonging to the intervention arm of the study. These participants from the on-site point-of-care (O-PoC) arm will be asked to reflect on their experience of receiving regular asymptomatic testing, COVID-19 education and PPE, and supportive counseling at Fortune Society from the CHW. These one-time semi-structured interviews will be conducted during the one-year participation.

Equal clusters of participants interviewed at each major 3-month milestone in the study (e.g. 5 participants interviewed at each 3-month mark). Participants who complete a qualitative interview will be compensated $40 and an additional $5.80 to cover the cost of roundtrip transportation to and from the study visit if opting for an in-person visit.

Analytic plan
Participant interviews will be audio-recorded, digitally stored on an encrypted password-protected folder within box.com, a HIPAA-compliant web-based data system, and professionally transcribed without participant identifiers. We will use thematic analysis to identify key themes relating to uptake of COVID-19 testing and mitigation factors. *Initial review of data*: In an iterative process, Dr. Akiyama and team will read all interview transcripts and record concepts that emerge upon repeated readings. *Developing a coding scheme*: The coding scheme will be based on the interview data and the goals of this project. The codebook will be discussed and agreed upon by the entire research team. Any necessary changes will be made by consensus. *Coding*: Using the codebook, two research team members will code data independently. The research team will meet, discuss codes, and, if there are any inconsistencies, will come to a consensus agreement as to application of codes for each transcript. Using Dedoose software (SocioCultural Research Consultants, Los Angeles CA), codes will be applied to the dataset and data will be organized by code. Sorting: After data are sorted and main themes are identified, the research team will meet again to ensure that all concepts are properly represented and that thematic saturation has occurred. *Member checking*: Identified themes will be presented to study participants to elicit interpretation and additional insight

G. CHW Audio diary recordings

We will collect audio diaries from the CHW at the end of each day reflecting on their COVID-19 testing and education visits with participants. The CHW holds these visits with each participant enrolled in the on-site point-of-care (O-PoC) arm of the intervention once every 3 months, for a total of 5 visits throughout their 1 year of participation. The CHW will be informed not to state any identifying information about participants, particularly their name and date of birth. The audio diaries will supplement information collected in the CHW logs regarding COVID-19 education provided, including the CHW's own thoughts and reflections on the successes and limitations of the intervention. Audio diary entries will be recorded at the end of the workday in which the CHW had conducted one or more testing and education visits with participants. This recording schedule will be maintained until the study has concluded and the final participant has completed their last visit.

Analytic plan
Audio diaries interviews will be audio-recorded, digitally stored on an encrypted password-protected folder within box.com, a HIPAA-compliant web-based data system, and professionally transcribed without participant identifiers. We will use thematic analysis to identify key themes relating to COVID-19 education provided, including the CHW's own thoughts and reflections on the successes and limitations of the intervention. *Initial review of data*: In an iterative process, Dr. Akiyama and team will read all interview transcripts and record concepts that emerge upon repeated readings. *Developing a coding scheme*: The coding scheme will be based on the interview data and the goals of this project. The codebook will be discussed and agreed upon by the entire research team. Any necessary changes will be made by consensus. *Coding*: Using the codebook, two research team members will code data independently. The research team will meet, discuss codes, and, if there are any inconsistencies, will come to a consensus agreement as to application of codes for each transcript. Using Dedoose software (SocioCultural Research Consultants, Los Angeles CA), codes will be applied to the dataset and data will be organized by code. Sorting: After data are sorted and main themes are identified, the research team will meet again to ensure that all concepts are properly represented and that thematic saturation has occurred. *Member checking*: Identified themes will be presented to study participants to elicit interpretation and additional insight

Ethical considerations

A. Human subjects

The investigators are committed to the protection of the rights of all participants in the proposed research, in accordance with U.S., Albert Einstein College of Medicine regulations. Before the initiation of any human subjects research, the study (including protocol and informed consent) will be submitted for review and approval by all applicable Institutional Review Boards (IRBs) at collaborating institutions. This will include ethics committees at Albert Einstein College of Medicine (Einstein) and University of Bristol. These IRBs have a Federal Wide Assurance (FWA) and are registered with the U.S. Department of Health and Human Services (DHHS) Office of Human Research Protection (OHRP). Recruitment at Fortune will be overseen by the Einstein IRB. The Principal Investigator of this application (Dr. Akiyama) in collaboration with the key personnel will help ensure the following:

- Education for all study staff on the protection of human subject participants; all investigators and study staff will be required to complete and maintain CITI (Collaborative Institutional Training Initiative) training (www.citiprogram.org) accreditation;
- Verify that informed consent was correctly administered for all participants by reviewing signed copies of consent forms that have been approved by the IRBs;
- Review the study procedures to ensure that any required protocol amendments have been approved by the IRBs and are appropriately conveyed to the study teams;
- Monitor adverse events and ensure all events are appropriately reported to the IRBs, and that participants experiencing adverse events have received appropriate medical care, if necessary;
- Verify that any events resulting in a breach of confidentiality have been reported and documented;
- Review procedures for storing study data; ensure that information related to the study is kept confidential, and that only the necessary staff has access to study information.

B. Risks

This study is associated with minor risks as the only study-related intervention is discomfort with the nasal swab study visits. Additional risks associated with study participation include the inconvenience of attending research visits and threats to confidentiality.

Inconvenience of attending study visits: The study requires 5 study visits for over 12 months. We will provide compensation for study participation and inconvenience. Participants will receive $40 for each completed study visit as well as an additional $5.80 to cover the cost of roundtrip transportation to and from the study visit if opting for an in-person visit. Phones will be provided to individuals who do not have a smartphone or one that is compatible with the Ethica software, which we will use to measure access to SARS-CoV-2 testing and healthcare services, ability to socially distance, and knowledge translation from public health messaging, the web-based questionnaires will be sent through this application. For those opting for in-person visits we will attempt to have study visits coincide with the participant's regular visits to Fortune for their regular services, although participation in the study will require additional time spent with research staff. Participants will receive $10 for each of the 26 web-based surveys they complete as well. Participants will also be told that they may withdraw from the study, if they so choose, at any point, without any impact on their access to healthcare or access to Fortune Society's services.

Retention procedures: To aid in retention, we have 4 additional incentives:

1. Participants will receive an additional $5 for completing a 4-week check-in with the study coordinator to confirm/update their contact information.
2. Participants will receive $50 for completing BOTH the Month 3 and Month 6 visits (to be paid at the completion of the Month 6) and an additional $50 for completing BOTH the Month 9 and Month 12 visits (to be paid at the completion of the Month 12).
3. Participants will receive up to $40 (in $5 increments) in retention incentives for contacting MOSAIC staff and providing updated locator information between in-person visits. They will only be compensated for one contact per month between each in-person visit.

4. If the MOSAIC team is unable to establish contact with the participant for their study visit, we may incentivize their emergency contacts to help us get back in touch with them. We will not share any of personal information or details about the nature of the study. Contacts will receive $25 via electronic giftcard if they are able to successfully reconnect the participant with the MOSAIC team and the participant completes the study visit they were being contacted for. There will only be one compensation per study visit. Participants will have the option to state whether or not they would like this person to receive compensation.

Nasal specimen collection: Participants will have nasal samples collected at each O-PoC visit, (0, 3, 6, 9 and 12 months). These samples will be used to assess for current SARS-CoV-2 infection at these five time-points. Nasal collection may cause some discomfort where the swab enters the nasal cavity.

Onsite tests will be performed by CHWs under a CLIA waiver. Although CLIA-waived tests must be simple and have a low risk for erroneous results, this does not mean that waived tests are completely error-proof. Errors can occur at any point in the testing process, particularly when the manufacturer's instructions are not followed and when testing personnel are not familiar with all aspects of the test system. Additionally, some waived tests have potential for serious health impacts if performed incorrectly. For example, results from waived tests can be used to adjust medication dosages, such as prothrombin time testing in patients undergoing anticoagulant therapy and glucose monitoring in diabetics. With this in mind, onsite nasal sample collection will be performed under the supervision of the CHWs who have been trained by Cepheid. To ensure CHWs are following testing protocols correctly, the Einstein/Montefiore Project Coordinator will also perform quality assurance every week for the first month and every 4 weeks. Though we do not anticipate and will minimize this risk of tests not being performed optimally as describe above, there is a chance that the RT-PCR is administered incorrectly despite initial training and follow-up. However, since we will not utilize the SARS-CoV-2 tests results to make decisions surrounding medical care (only education and patient navigation), we do not anticipate that an incorrectly administered test will cause serious health impacts. CLIA waived, point-of-care testing for HIV and hepatitis C virus are already performed at Fortune Society locations by Fortune staff. This study will operate under a similar principle. All participants will be made aware of the signs and symptoms of severe COVID-19 disease and will be counseled to seek the appropriate care should they develop such symptoms. Onsite nasal collection will be conducted in private rooms at Fortune Society. CHWs will outfitted in proper PPE per CDC guidelines including face masks, surgical gloves, hand sanitizer and disinfectant wipes, and disposable surgical gowns when observing and/or conducting the nasal collection.

Confidentiality: Because this project focuses on COVID-19 among CLI, confidentiality is a major concern. We will be collecting personal information from participants to facilitate follow up and will be asking questions about sensitive data including health and COVID-19 exposure. Data from Program Coordinator logs and healthcare records will be de-identified and entered into REDCap, a secure HIPAA-compliant web-based data system. To maintain strict confidentiality of all data we will use separate processes for ID-based and name-based files and seek a Certificate of Confidentiality. Our procedures to maintain confidentiality are addressed below.

Protection against risks: Questioning about criminal activity and substance abuse requires special sensitivity to issues of confidentiality. Participant confidentiality will be carefully protected. We will institute the following processes to ensure confidentiality is maintained:

1. We will be using Ethica and REDCap for data collection and we will create a "name-based" system and "ID-based" system that will remain separate. In the name-based system, all documents that have patient identifiers will be filed together. Some of these documents will have participants' signatures (e.g. consent forms) and others will have personal information (e.g. locator forms). In the ID-based system, all documents that do not include identifying information or signatures will use participants' IDs (rather than names) and will be filed together. All forms will contain either participants' names or

their study IDs, but not both. There will only be one electronic document that links participants' names to their study IDs, which will be stored on a password-protected computer.

   a. Data collected via the Ethica system will also be de-identified. The participant's Ethica ID will be maintained across acquisition methodologies, leading to a classic anonymized data collection system. Given that data on location is collected, the Ethica team informs participants as part of the consent process that while they will make every effort to ensure their anonymity, it is possible for researchers to learn it, and requests that participants do not publish any data which might be individually identifying (such as a map of their daily routine) without their express written consent. The research team agrees to not intentionally seek to re-identify the data in efforts to identify the participant.
   b. REDCap, which uses HIPAA compliant systems, with end to end encryption, will be used to collect participant responses to study questionnaires, as well as enter and store Program Coordinator logs and healthcare records. Additionally, REDCap allows us to assign individuals different levels of access to the system and data and allows monitoring of data access and flags/rules can be created for any potential suspicious activity (e.g. a user accessing the system outside expected working hours).
   c. Data collection via phone carrier may also be enabled in some instances. At enrollment, participants who receive a study phone will be asked if they would like to share their location with the SC. This will not be used to continuously check participants' whereabouts. The study team will only access the shared location feature in the event a participant contacts the study to inform them their phone has been lost or stolen. Participants can turn their shared location services on and off as they choose. The research team agrees to not intentionally seek to identify a participants' whereabouts.

2. We will obtain a Certificate of Confidentiality to protect participants' sensitive information.
3. Letters and/or phone messages that are left for participants (to schedule research visits) will not include any personal identifying information, and will not mention criminal justice involvement or the study directly.
4. Study records will be kept in locked files and/or within limited access, code-protected computer files, available only to the investigators and study personnel.
5. Publication or presentation of study results will not identify subjects by name. In addition to addressing issues of confidentiality, it is crucial that we protect both peer mentors and participants from emotional distress that may occur during any of the intervention or research activities. During interviews we will ask sensitive questions about behaviors such as drug use. Members of the research team conducting the individual interviews are trained to be aware of and immediately address emotional distress. When participants become distressed, the member of the research team will immediately call the PI, Dr. Akiyama. Distressed participants will be referred to medical/psychiatric attention at their community health center or the emergency department (depending on severity).

In addition to interviews, the primary study procedures involve specimen collection. CHWs have direct experience working with this population and with public health-related research studies. Onsite nasal collection will be performed under the supervision of CHWs who agree to serve as testers and receive specific training from Cepheid to minimize discomfort and ensure safe specimen collection. Each subject will receive a unique study identification number. All research data and specimens will be labeled only with this number and contain no other individual identification. Only the written informed consent forms will have identifying information on them. Subjects' names and study ID numbers will be linked in a single, password protected computer file that will be kept on a computer which can only be accessed by senior project staff. This link between the subject and the research study will be destroyed 6 months after the end of the study. Study records will be kept in locked files and/or within limited access, password-protected computer files, available only to the investigators and study personnel. Publication or presentation of study results will not identify subjects by name. The investigators have extensive experience in the performance of similar studies among justice-involved individuals people living with infectious diseases.

C. Benefits

For individual subjects who participate in this study, the benefit will be the knowledge that they are contributing to the understanding of SARS-CoV-2 testing and mitigation among justice-involved individuals. Participants may also benefit from increased uptake and receipt of SARS-CoV-2 test results and mitigation measures. The risk/benefit ratio for this study is low, as the research procedures involve minimal risks (primarily nasal collection) and the importance of knowledge gained about COVID-19 transmission should be significant. There will be no expense to participants for participation in this study.

D. Informed consent

Active study recruitment will be employed for this study. During a regular client encounters (telephone and in-person), Fortune case workers will introduce the study to potential participants and actively refer those interested in participating to the study team for evaluation and enrollment. Due to the COVID-19 pandemic, many services are currently being provided telephonically, but Fortune's community sites are currently being reopened. If a client expresses interest and gives permission, the case worker will refer them to the SC for study screening over the phone. The SC will inform referred patients of the purpose of screening (to identify eligibility for the study); how long screening will take; their choice to participate; that their access to Fortune Society's services will not be affected by a choice not to participate; and that there will be no cost, compensation, or benefit to them of the screening. Potential study subjects will provide their verbal informed consent. Answers will be recorded anonymously. Waiver of written informed consent for the screening portion of the research will be sought from the IRB, since screening will consist only of an interview and review of the patient's SARS-CoV-2 history. If a potential subject is willing to participate, he or she will provide informed consent at the time of enrollment and prior to the administration of any study measures. If conducted remotely, verbal consent will be obtained in place of written consent to comply with COVID-19 precautions, *a strategy that we are currently using that has been sanctioned by the Einstein IRB due to COVID-19 for other research studies*. Verbally-consented participants will be mailed the informed consent document to sign with a pre-stamped return envelope to mail back the signed informed consent. If the consent and enrollment visit is conducted in-person, written informed consent will be obtained. During the consent process, the SC will review the elements of consent with subjects, including: that this is a research study; a description of the study procedures, including follow-up tracking and contacts; the risks, discomforts and benefits of the study; their choice to not participate in the study; the purpose and duration of the study; confidentiality; and that participation is voluntary. Study enrollment and participation will not interrupt or interfere with participants' care. Informed consent will be conducted prior to collecting any information with personal identifiers (e.g. locator form or medical record release forms), administering the baseline REDCap interview, and randomization to an intervention arm. Regarding follow-up and tracking, the SC will also inform individuals that if they do enroll in the study but are lost-to-follow-up, the research team may utilize the public NYC and/or NYS look up system to determine if the participant has been re-incarcerated.

a. Disenrollment

If a participant is unwilling to comply with all study procedures and availability for the duration of the study, this may be grounds for disenrollment. This may include: (1) When Fortune Society has notified the study team that the participant is no longer permitted on-site at Fortune's locations; (2) A participant moves out of the NYC area (one of the five boroughs) during the course of their participation in the study; or (3) A participant exhibits disruptive behavior or acts inappropriately toward the study staff. If the participant isn't following the study procedures, this would be grounds for removal from the study.

In the event a participant may need to be disenrolled, the research team will discuss together the benefits and risks of retaining the participant. If it is decided the participant will be disenrolled, a member of the research team will let the participant know that Dr. Akiyama will be contacting them to discuss the study. Dr. Akiyama will attempt to call the participant up to 3 times, and will leave a voicemail if contact is not established during the call. If the calls are not successful, the same member of the research team will send a text message to the participant informing them they are no longer enrolled with the reason why. Once the

participant has been informed they will be disenrolled from ethica and will not longer be able to complete any of the study activities. We will document all disenrollments in our adverse event log

E. Vulnerable population

We will not include fetuses, neonates, pregnant women, prisoners, or institutionalized individuals in the proposed research. The research team has extensive experience working with and enrolling justice-involved individuals into infectious disease research studies.

Data Safety and Monitoring Plan

The purpose of our Data and Safety Monitoring Plan is to ensure the safety of study participants and the validity of data, in compliance with the National Institutes of Health requirement of Data and Safety Monitoring for Clinical Trials. In this intervention, we will randomize 250 CLI in blocks of 4-8, with 1:1 allocation of O-PoC vs. enhanced referral to O-PoC or an enhanced referral to offsite community testing. This section outlines the essential elements of the Data and Safety Monitoring Plan for the clinical trial.

A. Data and Safety Monitoring Board (DSMB)

We will establish a Data and Safety Monitoring Board (DSMB) for the proposed study. This committee will be comprised of clinicians from Albert Einstein College of Medicine unaffiliated with the research study. The DSMB will meet prior to enrollment and vote on approval of the protocol prior to initiation and the minutes of the meeting will be submitted to the IRB within 30 days. It will review safety and trial progress and provide advice with respect to study continuation, modification, and/or termination. We will email reports to DSMB members every six months summarizing the number of participants screened, enrolled, and lost to follow up; and all adverse events. The DSMB will meet prior to enrollment, every 6 months, at end of study for review of data and findings for future intervention proposal development and as needed in-person or via conference call.

<u>Members and affiliation:</u> We will establish a DSMB for the proposed study. This committee will include researchers at the Albert Einstein College of Medicine unaffiliated with the study. We have chosen board members based on their scientific and clinical experience and expertise in infectious disease prevention and treatment and design of randomized controlled trials.

<u>Frequency of meetings.</u> Every 6 months, reports will be emailed to DSMB members summarizing the number of participants screened, enrolled, and lost to follow up; all adverse events; all pregnancies; and all protocol deviations. The DSMB will meet every 6 months and as needed in-person or via conference call.

<u>Conflict of interest.</u> The DSMB will be comprised of individuals who are unaffiliated with the study, and who have no financial interests related to the research or its sponsor.
Protection of confidentiality. Measures to ensure the confidentiality of research participants is outlined above. Monthly DSMB reports will not identify participants by name. Should IRB or DSMB members request to review study records, we will request that all protected health information be treated confidentially.

<u>Monitoring activities (initial and ongoing study review).</u> The DSMB will meet and vote on approval of the protocol prior to initiation and the minutes of the meeting will be submitted to Einstein IRB within 30 days. It will review safety and trial progress and provide advice with respect to study continuation, modification, and/or termination.

<u>Communication plan to IRB and NIH.</u> In accordance with Einstein IRB regulations, the DSMB will record minutes of all meetings. The minutes will include the following: 1) attendance, 2) summary of the

discussion, and 3) findings, (e.g., research may begin or continue, recruitment is halted, actions needed to re-open recruitment, etc.). The DSMB will send the investigator the IRB all minutes.

If the DSMB concludes that the protocol should continue, unmodified, the DSMB will send the investigator and the IRB the minutes, and no further action will be taken. If the DSMB concludes that changes to the protocol and/or the informed consent are required, but recruitment may continue, the PI will submit an amendment to implement the required changes for review by the IRB. If the DSMB concludes that recruitment should be stopped: 1) the DSMB will send the investigator the minutes with directive to suspend recruitment immediately; 2) the DSMB and IRB will copy each other on all written and electronic communications; 3) the IRB Chair or designee will review the DSMB recommendations, and if in agreement, 4) the IRB will notify the investigator, in writing, affirming the DSMB action, and directing the investigator to submit an amendment to implement the required changes; and 5) the PI will submit an amendment to implement the required changes for review by the IRB.

Any serious adverse event, whether or not it is related to study medications, will be reported to the DSMB, the IRB, and the NIH Program Officer within 5 business day by phone, email or fax. All adverse events will be compiled and reported in summary form every month and at the conclusion of the study. Unanticipated (non-serious) problems will be documented on the Einstein IRB Reportable Event Form, and reported to the IRB within 5 days. Serious adverse events will be reported to the IRB within 5 days via the Reportable Event Form on through the IRBManager web portal. A summary of the SAEs that occurred during the previous year will be included in the annual progress report to the IRB and to NIH. Dr. Akiyama will report all DSMB recommendations and IRB actions, including protocol amendments in summary form to NIH on an annual basis as part of the progress repo

B. Adverse Event Monitoring

Collection of AEs and SAEs: Because participants are participating in SARS-CoV-2 testing intervention, participants are likely to incur no more than minimal risk of AEs. However, if AEs occur, they may include discomfort with personal questions, discomfort with conducting or receiving an nasal swab, and results of a SARS-CoV-2 PCR test. AEs will be collected by members of the research team as they conduct research visits and will be reported to Dr. Akiyama either immediately (for serious AEs) or during regular weekly project meetings (for non-serious AEs). Serious AEs could include death, life-threatening adverse reaction, inpatient hospitalization, persistent disability, or an event jeopardizing the participant’s health.

Reporting of AEs and SAEs: Consistent with the policies of the Einstein IRB, all adverse events (AEs) will be reported to Dr. Akiyama, who will maintain a log of AEs and will decide about the need to report to the IRB. All serious adverse events (SAEs) will be logged and reported to the DSMB and the Einstein IRB using the within 5 days via the Reportable Event Form on through the IRB Manager web portal. As per Einstein IRB policy, anticipated adverse event, such as psychological distress, do not require individual reporting to the IRB, but will be compiled and reported to the DSMB, Einstein IRB, and the NIH, in summary form every 6 months and at the conclusion of the study. Unanticipated (non-serious) problems will be documented on the Einstein IRB Reportable Event Form, and reported to the IRB within 5 days. All deaths will be reported to the DSMB, IRB, and NIH within 48 hours.

Management of SAEs or other study risks: Dr. Akiyama will be contacted by cell phone if any study participants become psychologically distressed. Dr. Akiyama (a board-certified infectious disease specialist) will make a clinical assessment or the participants’ health and safety. Depending on severity of the problem, participants may be escorted to either Montefiore’s community health center or emergency department. Participants may be referred for individual medical or psychiatric attention at Montefiore’s community health center or the emergency department (depending on severity). We have also set up several precautions to prevent breach of confidentiality and these have been described above.

C. Interim Analyses and Early Study Termination

The investigative team and the DSMB will conduct interim monitoring of adverse events every six months, and as necessary. A decision to stop the study may be made at any time that the research team and DSMB agree that an unacceptable type and/or frequency of adverse events has been observed.
Interim analysis of efficacy data: Interim analysis of the data will be conducted annually starting in year 2. If the results show overwhelming, statistically significant differences in SARS-CoV-2 testing and linkage-to-care between study arms, the study will be stopped, or only the arm that has not shown benefit. Dr. Akiyama will report the decision to terminate the study or change protocol to the DSMB, IRB and NIH within 48 hours of this determination. He will submit a narrative description of the reasons for early termination of the study to the IRB and NIH within 10 days.

D. DSM PLAN ADMINISTRATION

Responsibility for data and safety monitoring: The Principal Investigator, Dr. Akiyama, will be responsible for monitoring the safety and efficacy of this trial, executing the data and safety monitoring plan, and complying with the reporting requirements. The DSMB will vote on approval of the protocol prior to initiation of the RCT and submit meeting minutes to the IRB within 30 days. The DSMB will review safety and trial progress and provide advice with respect to study continuation, modification, and/or termination.

Frequency of DSM reviews: We will conduct continuous, close monitoring by study staff and investigators, with prompt identification and reporting of adverse events. Information about all unanticipated and serious adverse events will be reported as described above. The Principal Investigator will provide a summary of the data and safety monitoring report to the Program Officer at NIH on an annual basis as part of the progress report. The DSMB will meet every 6 months and as needed in-person or via video-conference call.

Content of DSM report: Dr. Akiyama will provide a summary of study activities to the DSMB every 6 months. This will include the number of participants screened, enrolled, and lost to follow up; all adverse events; and all protocol deviations. Dr. Akiyama will provide a summary of the data and safety monitoring report to the Program Officer at NIH on an annual basis as part of the progress report. The data and safety monitoring report will include a brief description of the RCT, and a summary of baseline sociodemographic characteristics of participants enrolled to that point. The report will include a summary of the total number of participants screened, enrolled, and lost to follow up; and quality assurance or regulatory issues that have taken place in the past year; all actions or changes taken by the IRB with respect to the protocol; and all unanticipated adverse events (AEs) and Serious Adverse Events (SAEs). If applicable, the data safety and management report to NIH will also include the results of any efficacy data analyses conducted.

REFERENCES

1. Johns Hopkins University. COVID-19 Dashboard by the Center for Systems Science and Engineering (CSSE) at Johns Hopkins University (JHU). https://coronavirus.jhu.edu/map.html. Accessed December 1, 2020.
2. Akiyama MJ, Spaulding AC, Rich JD. Flattening the Curve for Incarcerated Populations - Covid-19 in Jails and Prisons. *N Engl J Med.* 2020;382(22):2075-2077.
3. Kinner SA, Young JT, Snow K, et al. Prisons and custodial settings are part of a comprehensive response to COVID-19. *Lancet Public Health.* 2020;5(4):e188-e189.
4. Lim YW, Andersen R, Leake B, Cunningham W, Gelberg L. How accessible is medical care for homeless women? *MedCare.* 2002;40(6):510-520.
5. Reinhart E, Chen D. Incarceration And Its Disseminations: COVID-19 Pandemic Lessons From Chicago's Cook County Jail. *Health Affairs.* 2020:10.1377/hlthaff.2020.00652.
6. USICH. *Connecting People Returning from Incarceration with Housing and Homelessness Assistance.* United States Interagency Council on Homelessness;2016.
7. Levanon Seligson A, Parvez FM, Lim S, et al. Public Health and Vulnerable Populations: Morbidity and Mortality Among People Ever Incarcerated in New York City Jails, 2001 to 2005. *J Correct Health Care.* 2017;23(4):421-436.
8. Lim S, Seligson AL, Parvez FM, et al. Risks of drug-related death, suicide, and homicide during the immediate post-release period among people released from New York City jails, 2001-2005. *Am J Epidemiol.* 2012;175(6):519-526.
9. Gu T, Mack JA, Salvatore M, et al. Characteristics Associated With Racial/Ethnic Disparities in COVID-19 Outcomes in an Academic Health Care System. *JAMA Netw Open.* 2020;3(10):e2025197.
10. Hawkins RB, Charles EJ, Mehaffey JH. Socio-economic status and COVID-19-related cases and fatalities. *Public Health.* 2020;189:129-134.
11. Federal Bureau of Prisons. https://www.bop.gov/about/statistics/statistics_inmate_ethnicity.jsp. Accessed November 1, 2020.
12. Puglisi L, Calderon JP, Wang EA. What Does Health Justice Look Like for People Returning from Incarceration? *AMA J Ethics.* 2017;19(9):903-910.
13. Wang EA, White MC, Jamison R, Goldenson J, Estes M, Tulsky JP. Discharge planning and continuity of health care: findings from the San Francisco County Jail. *Am J Public Health.* 2008;98(12):2182-2184.
14. Walsh-Felz D, Westergaard R, Waclawik G, Pandhi N. "Service with open arms": enhancing community healthcare experiences for individuals with a history of incarceration. *Health Justice.* 2019;7(1):20.
15. Valera P, Boyas JF, Bernal C, Chiongbian VB, Chang Y, Shelton RC. A Validation of the Group-Based Medical Mistrust Scale in Formerly Incarcerated Black and Latino Men. *Am J Mens Health.* 2018;12(4):844-850.
16. Kutnick AH, Leonard NR, Gwadz MV. "Like I Have No Choice": A Qualitative Exploration of HIV Diagnosis and Medical Care Experiences While Incarcerated and Their Effects. *Behav Med.* 2019;45(2):153-165.
17. Cutler DM, Summers LH. The COVID-19 Pandemic and the $16 Trillion Virus. *JAMA.* 2020;324(15):1495-1496.
18. So L, Smith G. In four U.S. state prisons, nearly 3,300 inmates test positive for coronavirus -- 96% without symptoms. https://www.reuters.com/article/us-health-coronavirus-prisons-testing-in/in-four-u-s-state-prisons-nearly-3300-inmates-test-positive-for-coronavirus-96-without-symptoms-idUSKCN2270RX. Accessed August 1, 2020.

19. The Marshall Project. A State-by-State Look at Coronavirus in Prisons. https://www.themarshallproject.org/2020/05/01/a-state-by-state-look-at-coronavirus-in-prisons. Accessed August 1, 2020.
20. Franco-Paredes C, Jankousky K, Schultz J, et al. COVID-19 in jails and prisons: A neglected infection in a marginalized population. *PLoS Negl Trop Dis.* 2020;14(6):e0008409.
21. Saloner B, Parish K, Ward JA, DiLaura G, Dolovich S. COVID-19 Cases and Deaths in Federal and State Prisons. *JAMA.* 2020;324(6):602-603.
22. Reinhart E, Chen DL. Incarceration And Its Disseminations: COVID-19 Pandemic Lessons From Chicago's Cook County Jail. *Health Aff (Millwood).* 2020;39(8):1412-1418.
23. American Civil Liberties Union. COVID-19 Model Finds Nearly 100,000 More Deaths Than Current Estimates, Due to Failures to Reduce Jails. https://www.aclu.org/sites/default/files/field_document/aclu_covid19-jail-report_2020-8_1.pdf. Accessed August 1, 2020.
24. Prison Policy Initative. https://www.prisonpolicy.org/blog/2020/05/14/jails-vs-prison-update/. Published 2020. Accessed July 11, 2020.
25. Mervosh S, Fernandez M. 'It's Like Having No Testing': Coronavirus Test Results Are Still Delayed. The New York Times. https://www.nytimes.com/2020/08/04/us/virus-testing-delays.html. Accessed October 30, 2020.
26. Schneider EC. Failing the Test - The Tragic Data Gap Undermining the U.S. Pandemic Response. *N Engl J Med.* 2020;383(4):299-302.
27. Ali S, Asaria M, Stranges S. COVID-19 and inequality: are we all in this together? *Can J Public Health.* 2020;111(3):415-416.
28. Huerto R, Goold SD, Newton D. Targeted Coronavirus Testing Is Essential For Health Equity. https://www.healthaffairs.org/do/10.1377/hblog20200611.868893/full/. Accessed November 1, 2020.
29. Schmitt-Groh S, Teoh K, Uribe M. Covid-19: Testing Inequality in New York City. http://www.columbia.edu/~mu2166/stu_covid19/slides_stu_covid19.pdf. Accessed November 15, 2020.
30. Rubin-Miller L, Alban C, Sullivan S, Artiga S. COVID-19 Racial Disparities in Testing, Infection, Hospitalization, and Death: Analysis of Epic Patient Data. Epic Health Research Network. https://www.ehrn.org/articles/covid-19-racial-disparities-in-testing-infection-hospitalization-death/. Accessed November 20, 2020.
31. Hill J, Barr L. No COVID-19 tests available for prisoners at center of New York outbreak, court documents show. ABC News. https://abcnews.go.com/Health/covid-19-tests-prisoners-center-york-outbreak-court/story?id=69969077. Accessed October 30, 2020.
32. Brown SR, Goldberg N. Audit of Brooklyn federal jail claims lack of COVID-19 tests may have masked size of outbreak. New York Daily News. https://www.nydailynews.com/new-york/ny-brooklyn-mdc-covid-19-testing-shortage-oig-report-20201110-mniz4ygxnbbglhrjotaxin4tfq-story.html. Accessed.
33. Centers for Disease Control. Interim Considerations for SARS-CoV-2 Testing in Correctional and Detention Facilities. https://www.cdc.gov/coronavirus/2019-ncov/community/correction-detention/testing.html. Accessed November 10, 2020.
34. Servick K. Pandemic inspires new push to shrink jails and prisons. https://www.sciencemag.org/news/2020/09/pandemic-inspires-new-push-shrink-jails-and-prisons. Accessed October 30, 2020.
35. Mizumoto K, Kagaya K, Zarebski A, Chowell G. Estimating the asymptomatic proportion of coronavirus disease 2019 (COVID-19) cases on board the Diamond Princess cruise ship, Yokohama, Japan, 2020. *Euro Surveill.* 2020;25(10).

36. Solomon L, Montague BT, Beckwith CG, et al. Survey finds that many prisons and jails have room to improve HIV testing and coordination of postrelease treatment. *Health Aff (Millwood).* 2014;33(3):434-442.
37. Ott IM, Strine MS, Watkins AE, Boot M, Kalinich CC, Harden CA. Simply saliva: stability of SARS-CoV-2 detection negates the need for expensive collection devices. *medRxiv 2020080320165233; doi: https://doiorg/101101/2020080320165233.*
38. National Institute of Corrections. THE ROLE OF SCREENING AND ASSESSMENT IN JAIL REENTRY. https://www.urban.org/sites/default/files/publication/25876/412669-The-Role-of-Screening-and-Assessment-in-Jail-Reentry.PDF. Accessed November 1, 2020.
39. Nelson J. House Appropriations Committee Approves '21 Funding for DOJ Programs. https://csgjusticecenter.org/house-appropriations-committee-approves-21-funding-for-doj-programs/. Accessed August 1, 2020.
40. Bannan CL, Lynch PA, Conroy EP, et al. Point-of-care testing for HIV in an Irish prison setting: results from three major Irish prisons. *Int J STD AIDS.* 2016;27(11):950-954.
41. Beckwith CG, Atunah-Jay S, Cohen J, et al. Feasibility and acceptability of rapid HIV testing in jail. *AIDS Patient Care STDS.* 2007;21(1):41-47.
42. Beckwith CG, Kurth AE, Bazerman LB, et al. A pilot study of rapid hepatitis C virus testing in the Rhode Island Department of Corrections. *J Public Health (Oxf).* 2016;38(1):130-137.
43. Wolters F, van de Bovenkamp J, van den Bosch B, et al. Multi-center evaluation of cepheid xpert(R) xpress SARS-CoV-2 point-of-care test during the SARS-CoV-2 pandemic. *J Clin Virol.* 2020;128:104426.
44. Cepheid. https://www.cepheid.com/coronavirus. Accessed November 15, 2020.
45. The United States Food and Drug Administration. https://www.accessdata.fda.gov/cdrh_docs/clia_waivers/K180218.pdf. Accessed November 15th, 2020.
46. Frank JW, Wang EA, Nunez-Smith M, Lee H, Comfort M. Discrimination based on criminal record and healthcare utilization among men recently released from prison: a descriptive study. *Health Justice.* 2014;2:6.
47. Wang EA, Hong CS, Samuels L, Shavit S, Sanders R, Kushel M. Transitions clinic: creating a community-based model of health care for recently released California prisoners. *Public Health Rep.* 2010;125(2):171-177.
48. Fox AD, Anderson MR, Bartlett G, Valverde J, Starrels JL, Cunningham CO. Health outcomes and retention in care following release from prison for patients of an urban post-incarceration transitions clinic. *J Health Care Poor Underserved.* 2014;25(3):1139-1152.
49. Wang EA, Hong CS, Shavit S, Sanders R, Kessell E, Kushel MB. Engaging individuals recently released from prison into primary care: a randomized trial. *Am J Public Health.* 2012;102(9):e22-29.
50. Collica-Cox K. Female Offenders, HIV Peer Programs, and Attachment: The Importance of Prison-Based Civilian Staff in Creating Opportunities to Cultivate Prosocial Behaviors. *Int J Offender Ther Comp Criminol.* 2018;62(2):524-550.
51. Collica-Cox K. Counting down: HIV prison-based peer education programs and their connection to reduced disciplinary infractions. *Int J Offender Ther Comp Criminol.* 2014;58(8):931-952.
52. Cunningham WE, Weiss RE, Nakazono T, et al. Effectiveness of a Peer Navigation Intervention to Sustain Viral Suppression Among HIV-Positive Men and Transgender Women Released From Jail: The LINK LA Randomized Clinical Trial. *JAMA Intern Med.* 2018;178(4):542-553.
53. Centers for Disease Control. https://www.cdc.gov/coronavirus/2019-ncov/global-covid-19/home-based-care.html. Accessed November 15, 2020.
54. Ballard M, Bancroft E, Nesbit J, et al. Prioritising the role of community health workers in the COVID-19 response. *BMJ Glob Health.* 2020;5(6).

55. Bhaumik S, Moola S, Tyagi J, Nambiar D, Kakoti M. Community health workers for pandemic response: a rapid evidence synthesis. *BMJ Glob Health.* 2020;5(6).
56. Partners in Health. https://www.pih.org/pages/coronavirus-full-response. Accessed November 15, 2020.
57. Peretz PJ, Islam N, Matiz LA. Community Health Workers and Covid-19 - Addressing Social Determinants of Health in Times of Crisis and Beyond. *N Engl J Med.* 2020;383(19):e108.
58. Counihan H, Harvey SA, Sekeseke-Chinyama M, et al. Community health workers use malaria rapid diagnostic tests (RDTs) safely and accurately: results of a longitudinal study in Zambia. *Am J Trop Med Hyg.* 2012;87(1):57-63.
59. Boyce MR, Menya D, Turner EL, Laktabai J, Prudhomme-O'Meara W. Evaluation of malaria rapid diagnostic test (RDT) use by community health workers: a longitudinal study in western Kenya. *Malar J.* 2018;17(1):206.
60. Juneau C, Pueyo T, Bell M, Gee G, Collazzo P, Potvin L. Evidence-Based, Cost-Effective Interventions To Suppress The COVID-19 Pandemic: A Systematic Review. https://www.medrxiv.org/content/10.1101/2020.04.20.20054726v2. Accessed November 1, 2020.
61. Centers for Disease Control. Scientific Brief: Community Use of Cloth Masks to Control the Spread of SARS-CoV-2. https://www.cdc.gov/coronavirus/2019-ncov/more/masking-science-sars-cov2.html. Accessed November 20, 2020.
62. Xiao J, Shiu EYC, Gao H, et al. Nonpharmaceutical Measures for Pandemic Influenza in Nonhealthcare Settings-Personal Protective and Environmental Measures. *Emerg Infect Dis.* 2020;26(5):967-975.
63. Lyu W, Wehby GL. Community Use Of Face Masks And COVID-19: Evidence From A Natural Experiment Of State Mandates In The US. *Health Aff (Millwood).* 2020;39(8):1419-1425.
64. Greenhalgh T, Schmid MB, Czypionka T, Bassler D, Gruer L. Face masks for the public during the covid-19 crisis. *BMJ.* 2020;369:m1435.
65. Brainard J, Jones N, Lake I, Hooper L, Hunter PR. https://www.medrxiv.org/content/10.1101/2020.04.01.20049528v1. Accessed November 20, 2020.
66. The Institute for Health Metrics and Evaluation. https://covid19.healthdata.org/united-states-of-america?view=daily-deaths&tab=trend. Accessed November 20, 2020.
67. Jefferson T, Del Mar CB, Dooley L, et al. Physical interventions to interrupt or reduce the spread of respiratory viruses. *Cochrane Database Syst Rev.* 2020;11:CD006207.
68. Matrajt L, Leung T. Evaluating the Effectiveness of Social Distancing Interventions to Delay or Flatten the Epidemic Curve of Coronavirus Disease. *Emerg Infect Dis.* 2020;26(8):1740-1748.
69. MacIntyre CR, Cauchemez S, Dwyer DE, et al. Face mask use and control of respiratory virus transmission in households. *Emerg Infect Dis.* 2009;15(2):233-241.
70. Alsan M, Stantcheva S, Yang D, Cutler D. Disparities in Coronavirus 2019 Reported Incidence, Knowledge, and Behavior Among US Adults. *JAMA Netw Open.* 2020;3(6):e2012403.
71. Bandura A. Health promotion by social cognitive means. *Health Educ Behav.* 2004;31(2):143-164.
72. The National Reentry Resource Center. Reentry Services Directory. https://nationalreentryresourcecenter.org/reentry-services-directory/. Accessed November 14, 2020.
73. Frimpong JA, Shiu-Yee K, Tross S, et al. Bundling Rapid Human Immunodeficiency Virus and Hepatitis C Virus Testing to Increase Receipt of Test Results: A Randomized Trial. *Med Care.* 2020;58(5):445-452.
74. Frimpong JA, D'Aunno T, Perlman DC, et al. On-site bundled rapid HIV/HCV testing in substance use disorder treatment programs: study protocol for a hybrid design randomized controlled trial. *Trials.* 2016;17(1):117.
75. Rosenberg ES, Dufort EM, Blog DS, et al. COVID-19 Testing, Epidemic Features, Hospital Outcomes, and Household Prevalence, New York State-March 2020. *Clin Infect Dis.* 2020;71(8):1953-1959.

76. Jing QL, Liu MJ, Zhang ZB, et al. Household secondary attack rate of COVID-19 and associated determinants in Guangzhou, China: a retrospective cohort study. *Lancet Infect Dis.* 2020;20(10):1141-1150.
77. Briggs A. Estimating QALY losses associated with deaths in hospital (COVID-19). https://avalonecon.com/wp-content/uploads/2020/04/COVID-19-QALYs-v3.pdf. Accessed November 30, 2020.
78. Dinnes J, Deeks JJ, Adriano A, et al. Rapid, point-of-care antigen and molecular-based tests for diagnosis of SARS-CoV-2 infection. *Cochrane Database Syst Rev.* 2020;8:CD013705.
79. Pasomsub E, Watcharananan SP, Boonyawat K, et al. Saliva sample as a non-invasive specimen for the diagnosis of coronavirus disease 2019: a cross-sectional study. *Clin Microbiol Infect.* 2020.
