# Supplementary material for: Mitigation of COVID-19 through onsite testing and education among formerly incarcerated individuals (the MOSAIC study): an open-label, single-centre, randomised controlled trial
Source: Lancet Public Health. Author manuscript; Available in PMC 2026 Jul 7. (PMC13338770; doi:10.1016/S2468-2667(26)00093-9)
Supplement: 2 [file NIHMS2190773-supplement-2.pdf]

THE LANCET
Public Health

Supplementary appendix 2

This appendix formed part of the original submission and has been peer reviewed. We post it as supplied by the authors.

Supplement to: Akiyama MJ, Kaba-Diakite F, Dimaulaluan M, et al. Mitigation of COVID-19 through onsite testing and education among formerly incarcerated individuals (the MOSAIC study): an open-label, single-centre, randomised controlled trial. *Lancet Public Health* 2026; **11:** e457–67.

Table of Contents

Page 2. Supplementary Table 1. Per Protocol Analyses: Regressions of study arms (O-PoC vs. SoC) as related to complete SARS-CoV-2 tests during the 12-month study period.

Page 3. Supplementary Table 2. Protocol deviations and adverse events

Page 4. Supplementary Table 3. COVID-19 tests during follow-up

Page 5. Data and Safety Monitoring Board (DSMB)

Page 9. Interim analyses

Supplementary Table 1. Per Protocol Analyses: Regressions of study arms (O-PoC vs. SoC) as related to complete *SARS-CoV-2* tests during the 12-month study period.

Bivariate Logistic Regression		
**Dependent Variable**	**OR (95% CI)**	**p-value**
At least one complete *SARS-CoV-2* test during the 12-month study period	5.2 (2.7 – 9.8)	<0.001
**Bivariate Poisson Regression**		
	IRR (95% CI)	p-value
Number of complete *SARS-CoV-2* tests during the 12-month study	2.2 (1.7 – 2.8)	<0.001

**Supplementary Table 2. Protocol deviations and adverse events**	
	Count
**Deviation**	
Social media section of consent left unchecked	91
Consented with expired consent form	25
Date missing from study coordinator's signature on consent form	11
Monolingual Spanish speaker and signed English consent	3
Research team did not provide follow-up visit reminder	5
Enrolled more than 30 days after being screened	5
Enrolled beyond 90 days of release	4
Witness consent was used unnecessarily	4
Completed follow up visit outside visit window	3
Moved out of New York City*	1
SOC participant scheduled for OPoC test	1
**Adverse event**	
Incarcerated during study period	64
Disenrolled, no longer allowed onsite at the CBO	4
Expressed suicidal ideation to a member of the research team	3
Disenrolled, inappropriate behavior toward research team	2
Disenrolled, relocated to a different state	1
Withdrew self	1
Death (unrelated to study participation)	1

* Individual relocated from New York City to Long Island, New York. The research team agreed to allow continued participation given their proximity to New York City, their frequency of being in New York City for employment and court mandated services, as well as regular attendance at the partnering organization.

**Supplementary Table 3. COVID-19 tests during follow-up**			
	Total (n=614)	Onsite point-of-care testing and education group (n=327)	Standard of care group (n=287)
Do you think that you have or have had COVID-19 in the last 3 months?*			
Yes, confirmed by a positive test	12 (2.0%)	10 (3.1%)	2 (0.7%)
Yes, my own suspicions	6 (1%)	4 (1.2%)	2 (0.7%)
*self-reported at follow-up			

Data and Safety Monitoring Board (DSMB)

1. DSMB Charter

Leveraging community health workers to improve SARS-CoV-2 testing and mitigation among criminal justice-involved individuals accessing a corrections-focused community-based organization (MOSAIC)

Albert Einstein College of Medicine/ Montefiore Medical Center #2021-12976

This charter defines the roles and responsibilities of the Data and Safety Monitoring Board (DSMB) for MOSAIC which is funded by the National Institute on Minority Health and Health Disparities (NIMHD).

The DSMB will serve in accordance with the guidelines set forth in this charter. If changes to the charter are necessary, the DSMB reviews and affirms their agreement with the changes. Their concurrence will be noted in the DSMB meeting summary.

DSMB RESPONSIBILITIES

Generally, the first responsibility of the DSMB will be to approve the final protocol of the clinical study named above, so that the study can begin enrolling participants. After initial approval, and at periodic intervals during the course of the study, the DSMB responsibilities are to:

- Provide input to assist the investigator(s) in protecting the safety of the study participants;
- Provide input to the investigator(s) on major changes to the research protocol, informed consent documents and plans for data and safety monitoring;
- Provide input to the investigator(s) on the progress of the study, including periodic assessments of data quality and timeliness, participant recruitment, accrual and retention, participant risk versus benefit, performance of the study sites, and other factors that may affect study outcomes;
- Review areas of concern regarding the performance of individual sites and provide comment to the investigator(s) on actions to be considered regarding sites that perform unsatisfactorily;
- Consider factors external to the study when relevant information becomes available, such as scientific or therapeutic developments that may have an impact on the safety of the participants or the ethics of the study;
- Provide input to the investigator(s) on modification of the study protocol or possible early termination of the study because of attainment of study objectives, safety concerns, low likelihood of showing a benefit of the intervention, or inadequate performance (such as enrollment and retention problems);
- Review the interim analysis and determine if an unacceptable type and/or frequency of adverse events has been observed suggesting termination of the study (following concurrence of the DSMB);
- Provide input to the investigator(s) on the potential impact of ancillary studies on the integrity of the parent study;

MEMBERSHIP

The members have been appointed by the investigator(s). Members of the DSMB shall have no financial, scientific, or other conflict of interest with the study.

DSMB MEETINGS

The DSMB will meet prior to enrollment, every 6 months (or as deemed necessary), at end of study for review of data and findings for future intervention proposal development and as needed in-person or via conference call. A quorum of more than half of the DSMB members is required in order to convene a meeting of the DSMB.

Meetings are attended, when appropriate, by the principal investigator and members of their staff, as well as the study statistician. Meetings may be convened as conference calls or webinars, as well as in person. In special

circumstances, the meetings may also be conducted by email. An emergency meeting of the DSMB may be called at any time should questions of patient safety arise.

MEETING FORMAT

An appropriate format for DSMB meetings consists of an open, closed (if the DSMB is monitoring a study in which the investigators are masked in any way), and executive session. This format may be modified as needed.

Open Session

Members of the DSMB, the principal investigator and members of the steering committee, including the study biostatistician may attend the open session. Issues discussed will include the conduct and progress of the study, including patient recruitment, data quality, general adherence and toxicity issues, compliance with protocol, and any other logistical matters that may affect either the conduct or outcome of the study. Proposed protocol amendments will also be presented in this session. Patient-specific data and treatment group data may not be presented in the open session.

Closed Session

The closed session will be attended only by DSMB members, Project Director, and the unmasked study biostatistician. The discussion at the closed session is completely confidential. All materials from the closed session will be filed so that they are only accessible by the individuals in the closed session.

Analyses of outcome data are reviewed by intervention groups, including baseline characteristics, primary and secondary outcomes, adverse events, adherence and dropouts, and examination of any relevant subgroups.

Executive Session

The executive session will be attended by DSMB members only, who will discuss the information presented during the closed and open sessions and provide input on the continuation or termination of the study, protocol modification or other changes to the conduct of the study. The DSMB can be unmasked at any time if trends develop either for benefit or harm to the participants.

The DSMB will make a recommendation for either continuation or termination of the study.

Termination may be suggested by the DSMB at any time. Reasons for early termination include:

- Serious adverse effects in entire intervention group or in a dominating subgroup;
- Greater than expected beneficial effects;
- A statistically significant difference by the end of the study is improbable;
- Logistical or data quality problems so severe that correction is not feasible. Sound rationale for either decision (continuation or termination of the study) should be presented.

REPORTS TO THE DSMB

Reports will be prepared by the biostatistician on a semi-annual basis as decided by the investigator and the DSMB. The reports will be distributed to the DSMB after the meeting by email as well.

Open Session Report: This portion of the report provides information on study aspects such as accrual, baseline characteristics, and other general information on study status. This report is generally shared with all investigators involved with the clinical study. The reports contained in this section generally include:

- Comparison of Target Enrollment to Actual Enrollment by Month;
- Comparison of Target Enrollment to Actual Enrollment by Site;
- Overall Subject Status by Site, including: Subjects Screened, Enrolled, Active,
- Completed and Terminated;
- Demographic and Key Baseline Characteristics by Group;
- Treatment Duration for Subjects who Discontinue Therapy;
- Adverse Events/Serious Adverse Events by Site and Subject.

Closed Session Report: This report may contain data on study outcomes, including safety data. Data will be presented by treatment groups. The Closed Session Report is considered confidential and will be filed so that they are only accessible by the individuals in the closed session. Data files to be used for interim analyses will have undergone established editing procedures to the extent possible. This report should not be viewed by any members of the clinical study except the designated study statistician and Project Director.

DOCUMENTATION OF DSMB MEETINGS

Meeting summary

A formal summary containing the DSMB's input on the conduct of the study and their recommendation regarding continuation of the study will be prepared by the Project Director. Each DSMB summary will include the DSMB's recommendation regarding continuation or termination of the study. The DSMB meeting summary will not include unmasked data, discussion of the unmasked data, or any other confidential data. Once completed, the summary is sent to the DSMB members for their review and concurrence. When the summary is satisfactory to the DSMB members and concurrence with the summary is received, the summary will be sent to the PI. It is the responsibility of the PI to distribute the summary to all co-investigators.

It is the responsibility of the Project Director to assure that the DSMB summary is submitted to the Institutional Review Board (IRB) associated with the study.

CONFIDENTIALITY AND OBJECTIVITY

All materials, discussions and proceedings of the DSMB are completely confidential. Members and other participants in DSMB meetings are expected to maintain confidentiality. Closed session meeting materials should be destroyed in a secure manner (shredding) following each meeting.

In order to maintain their objectivity, DSMB members are expected not to discuss the study with the investigators except during DSMB meetings.

2. Summary of amendments proposed by the DSMB

There were minimal amendments proposed by the DSMB, all of which occurred within year 1 of the study regarding additional data collection measures. Each suggestion was incorporated:

1. Add question on SARS-CoV-2 testing during incarceration (prior to enrollment)
2. Add question to qualitative interviews on why participants came back for follow-up visits (to better understand retention)

DSMB members also proposed additional analyses to consider:

1. Quantitative:
    a. Compare early vs. later intervention periods to account for higher vs. lower COVID-risk phases
2. Qualitative:
    a. Identify key components of the CHW counseling and the nature of relationships built with participants
    b. Translate findings into hiring and training frameworks for future CHWs

Interim Analyses November 18, 2023

Section 1: Baseline characteristics and history of COVID-19 tests and vaccinations.

As of 11/18/2023, 218 participants were enrolled in the study and randomly assigned to the two study arms (O-POC=110; Standard of Care=108). The mean (SD) age was 42.5 (12.1), mean (SD) number of days incarcerated in the past 30 days before enrolled was 3.2 (6.3), and mean (SD) number of years incarcerated in lifetime was 12.9 years (11.9). Among them, 187 (85.8%) were males, 65 (29.8%) were Hispanic, and 133 (61.0%) were reported as homeless when enrolled. Before enrollment, 208 (95.4%) had had ever tested for COVID-19 and 76 (34.9%) were ever-tested positive. Majority of them (166, 76.2%) had received at least one dose of vaccine for COVID-19 and 81 (37.2%) had received at least one booster. None of the baseline characteristics and histories of COVID-19 tests, infections, and vaccination received were significantly different between the study arms (see Table 1).

Table 1. Chi-square tests (or Fisher exact tests) and t-tests for baseline characteristics and histories of COVID-19 tests, infection, and vaccination status by study arms (O-POC vs. Standard of Care).

Baseline Characteristics and histories of COVID-19 tests, infection, and vaccination received	Whole Sample (N=218) n (%) or mean (SD)	O-POC (N=110) n (%) or mean (SD)	Standard of Care (N=108) n (%) or Mean (SD)	p-value
Male, n (%)	187 (85.8)	92 (83.6)	95 (88.0)	0.361
Age, mean (SD)	42.5 (12.1)	43.4 (12.5)	41.6 (11.6)	0.261
Hispanic, n (%)	65 (29.8)	31 (28.2)	34 (31.5)	0.594
Homeless, n (%)	133 (61.0)	68 (61.8)	65 (60.2)	0.805
Number of days incarcerated in the past 30 days, mean (SD)	3.2 (6.3)	2.6 (5.8)	3.9 (6.8)	0.132
Number of years incarcerated in lifetime, mean (SD)	12.9 (11.9)	14.0 (12.1)	11.8 (11.8)	0.159
Ever tested for COVID-19 test, n (%)	208 (95.4)	105 (95.5)	103 (95.4)	0.976
Ever COVID-19 Positive, n (%)	76 (34.9)	38 (34.6)	38 (35.2)	0.921
Ever received COVID-19 Vaccines, n (%)	166 (76.2)	85 (77.3)	81 (75.0)	0.694
Ever received COVID-19 Booter, n (%)	81 (37.2)	44 (40.0)	37 (34.3)	0.381

Section 2: Test the effectiveness of an onsite PoC SARS-CoV-2 intervention.

H1: O-PoC (vs. SoC) will result in a greater proportion of tests performed and results received.

a. **Associations between study arms (O-POC vs. Standard of Care) and measures of COVID-19 tests between enrollment - months 3.**

As of 11/18/2023, we have months 3 COVID-19 testing data for 145 participants (O-POC=78, Standard of care=67). Table 2 presents Chi-square tests (or Fisher exact tests) for the associations between study arms (O-POC vs. Standard of Care) and measures of COVID-19 tests between enrollment - month 3. As compared to the Standard of Care arm, participants in the O-POC arm had significantly higher PCR-test rate, but lower antigen test rate. The differences in overall COVID-19 test rate and test results received rates were not significant between the two arms.

Table 2. Chi-square tests (or Fisher exact tests) for the associations between study arms (O-POC vs. Standard of Care) and measures of COVID-19 tests between enrollment - month 3.

	O-POC (N=78) n (%)	Standard of Care (N=67) n (%)	p-value
COVID-19 tests (yes/no)	72 (92.3)	58 (86.6)	0.258
COVID-19 test results received (yes/no)	72 (92.3)	57 (85.1)	0.166
PCR-tests (yes/no)	70 (89.7)	33 (49.3)	<0.001
Antigen-tests (yes/no)	14 (18.0)	29 (43.3)	<0.001

Table 3 presents results of logistic regression analyses of study arms (O-POC vs. Standard of Care) as related to measures of COVID-19 tests between enrollment – month 3 with/without controlling for baseline characteristics and history of COVID-19 tests and vaccinations. As compared to the Standard of Care arm, participants in the O-POC arm had significantly higher likelihood of having PCR-test, but lower likelihood of having antigen test. Overall, the differences in likelihood of having at least a COVID-19 test or receiving at least a test result were not significant between the two arms (with or without controlling for the covariates).

Table 3. Logistic regressions of study arms (O-POC vs. Standard of Care) as related to measures of COVID-19 tests between enrollment – month 3.

	**Bivariate Logistic Regression**		**Multiple Logistic Regression** [a]	
Dependent Variables	OR (95% CI)	p-value	AOR (95% CI)	p-value
COVID-19 tests (yes/no)	1.86 (0.63 – 5.54)	0.263	1.45 (0.44 – 4.80)	0.546
COVID-19 test results received (yes/no)	2.11 (0.72 – 6.14)	0.173	1.80 (0.56 – 5.75)	0.322
PCR-tests (yes/no)	9.02 (3.76 – 21.61)	<0.001	9.05 (3.67 – 22.34)	<0.001
Antigen-tests (yes/no)	0.29 (0.14 – 0.61)	0.001	0.23 (0.10 – 0.51)	<0.001

Note: [a] Age, gender, ethnicity, living situation, ever-tested, ever-vaccinated, and number of days incarcerated in the past 30 days before enrollment were controlled in the multiple logistic regressions.

Table 4 presents results of Poisson regression analyses of study arms (O-POC vs. Standard of Care) as related to number of COVID-19 tests between enrollment – month 3 with/without controlling for baseline characteristics and history of COVID-19 tests and vaccinations. As compared to the Standard of Care arm, participants in the O-POC arm had greater number COVID-19 tests, but not statistically significant.

Table 4. Poisson regressions of study arms (O-POC vs. Standard of Care) as related to number of COVID-19 tests between enrollment – month 3.

	**Bivariate Logistic Regression**		**Multiple Logistic Regression** [a]	
Dependent Variables	IRR (95% CI)	p-value	IRR (95% CI)	p-value
Number of COVID-19 tests	1.13 (0.89 – 1.44)	0.329	1.08 (0.84 – 1.38)	0.558

Note: [a] Age, gender, ethnicity, living situation, ever-tested, ever-vaccinated, and number of days incarcerated in the past 30 days before enrollment were controlled in the multiple Poisson regressions.

b. Associations between study arms (O-POC vs. Standard of Care) and measures of COVID-19 tests between months 3-6.

As of 11/18/2023, we have months 6 COVID-19 testing data for 119 participants (O-POC=60, Standard of care=59). Table 5 presents Chi-square tests (or Fisher exact tests) for the associations between study arms (O-POC vs. Standard of Care) and measures of COVID-19 tests between months 3-6.

Table 5. Chi-square tests (or Fisher exact tests) for the associations between study arms (O-POC vs. Standard of Care) and measures of COVID-19 tests between months 3-6.

	O-POC (N=60) n (%)	Standard of Care (N=59) n (%)	p-value
COVID-19 tests (yes/no)	55 (91.7)	43 (72.9)	0.007
COVID-19 test results received (yes/no)	55 (91.7)	42 (71.2)	0.004
PCR-tests (yes/no)	51 (85.0)	27 (45.8)	<0.001
Antigen-tests (yes/no)	12 (20)	14 (23.7)	0.622

Table 6 presents results of logistic regression analyses of study arms (O-POC vs. Standard of Care) as related to measures of COVID-19 tests between months 3–5 with/without controlling for baseline characteristics and history of COVID-19 tests and vaccinations.

Table 6. Logistic regressions of study arms (O-POC vs. Standard of Care) as related to measures of COVID-19 tests between 3-6 months.

	Bivariate Logistic Regression		Multiple Logistic Regression [a]	
Dependent Variables	OR (95% CI)	p-value	AOR (95% CI)	p-value
COVID-19 tests (yes/no)	4.09 (1.39 – 12.06)	0.011	4.54 (1.39 – 14.85)	0.012
COVID-19 test results received (yes/no)	4.45 (1.52 – 13.04)	0.002	5.29 (1.61 – 17.31)	0.006
PCR-tests between 3-6 months (yes/no)	6.72 (2.80 – 16.10)	<0.001	7.80 (3.03 – 20.07)	<0.001
Antigen-tests between 3-6 months (yes/no)	0.80 (0.34 – 1.92)	0.623	0.58 (0.21 – 1.63)	0.305

Note: [a] Age, gender, ethnicity, living situation, ever-tested, ever-vaccinated, and number of days incarcerated in the past 30 days before enrollment were controlled in the multiple logistic regressions.

Table 7 presents results of Poisson regression analyses of study arms (O-POC vs. Standard of Care) as related to number of COVID-19 tests with/without controlling for baseline characteristics and history of COVID-19 tests and vaccinations.

Table 7. Poisson regressions of study arms (O-POC vs. Standard of Care) as related to number of COVID-19 tests.

	Bivariate Logistic Regression		Multiple Logistic Regression [a]	
Dependent Variables	IRR (95% CI)	p-value	IRR (95% CI)	p-value
Number of COVID-19 tests between months 3-6	1.35 (0.99 – 1.84)	0.058	1.35 (0.99 – 1.86)	0.058
Number of COVID-19 tests between enrollment – month 6	1.27 (1.00 – 1.61)	0.05	1.24 (0.98 – 1.57)	0.081

Note: [a] Age, gender, ethnicity, living situation, ever-tested, ever-vaccinated, and number of days incarcerated in the past 30 days before enrollment were controlled

H2: O-PoC (vs. SoC) will be associated with increased mitigation behaviors.

Among the 218 MOSAIC participants, so far, mitigation behaviors were assessed for 215 participants. On average, there were 26 visits per participant (5526 total visits (time points)). On a scale of 1-100, mean (SD) masking, social distancing, and hand washing were 55.1 (36.2), 55.8 (34.8), and 73.4 (29.8), respectively. The three measures were significantly correlated with each other (r=0.48 -0.83, p<0.001).

Table 9 presents results of GEE models for the associations between study arms (O-POC vs. Standard of Care) and mitigation behaviors over time. As compared to the Standard of Care arm, participants in the O-POC arm had higher level of mitigation behaviors, but not statistically significant.

Table 9. GEE models of study arms (O-POC vs. Standard of Care) as related to mitigation behaviors.

	Bivariate GEE models		Multiple GEE models [a]	
Dependent Variables	beta (95% CI)	p-value	beta (95% CI)	p-value
Social distancing	3.39 (-5.03 – 11.81)	0.430	2.56 (-5.92 – 11.04)	0.555
Masking	8.16 (-0.88 – 17.20)	0.077	7.39 (-1.69 – 16.47)	0.111
Hand washing	2.72 (-4.61 – 10.06)	0.467	1.88 (-5.35 – 9.12)	0.610

Note: [a] Age, gender, ethnicity, living situation, ever-tested, ever-vaccinated, and number of days incarcerated in the past 30 days before enrollment were controlled.

c. Testing data from the Fortune

	Total	OPOC (on site)	SOC (off site)	P-Value
**Tested at 1st visit**	218	92/110 (84%)	58/108 (54%)	<0.001
**Tested at 2nd visit**	199	53/101 (52%)	30/98 (31%)	0.002
**Tested at 3rd visit**	174	48/85 (56%)	8/89 (9%)	<0.001
**Tested at 4th visit**	128	19/65 (29%)	8/63 (13%)	0.022
**Tested at 5th visit**	91	14/45 (31%)	2/46 (4%)	0.001 [a]

[a] exact tests

Interim Analyses June 27, 2024

Section1: Baseline characteristics and history of COVID-19 tests and vaccinations.

As of 06/17/2024, 250 participants were enrolled in the study and randomly assigned to the two study arms (O-POC=125; Standard of Care=125). The mean (SD) age was 42.0 (11.8), mean (SD) number of days incarcerated in the past 30 days before enrolled was 3.2 (6.3), and mean (SD) number of years incarcerated in lifetime was 12.8 years (11.6). Among them, 219 (87.6%) were males, 82 (32.8%) were Hispanic, and 155 (62.0%) were reported as homeless when enrolled. Before enrollment, 239 (95.6%) had had ever tested for COVID-19 and 85 (34.0%) were ever-tested positive. Majority of them (191, 76.4%) had received at least one dose of vaccine for COVID-19 and 95 (38.0%) had received at least one booster. None of the baseline characteristics and histories of COVID-19 tests, infections, and vaccination received were significantly different between the study arms (see Table 1).

Table 1. Chi-square tests (or Fisher exact tests) and t-tests for baseline characteristics and histories of COVID-19 tests, infection, and vaccination status by study arms (O-POC vs. Standard of Care).

Baseline Characteristics and histories of COVID-19 tests, infection, and vaccination received	Whole Sample (N=250) n (%) or mean (SD)	O-POC (N=125) n (%) or mean (SD)	Standard of Care (N=125) n (%) or Mean (SD)	p-value
Male, n (%)	219 (87.6)	107 (85.6)	112 (89.6)	0.337
Age, mean (SD)	42.0 (11.8)	42.8 (12.2)	41.2 (11.3)	0.279
Hispanic, n (%)	82 (32.8)	39 (31.2)	43 (34.4)	0.590
Homeless, n (%)	155 (62.0)	79 (63.2)	76 (60.8)	0.696
Number of days incarcerated in the past 30 days, mean (SD)	3.2 (6.3)	2.7 (6.0)	3.6 (6.5)	0.282
Number of years incarcerated in lifetime, mean (SD)	12.8 (11.6)	13.6 (11.7)	11.9 (11.4)	0.233
Ever tested for COVID-19 test, n (%)	239 (95.6)	120 (96.0)	119 (95.2)	0.758
Ever COVID-19 Positive, n (%)	85 (34.0)	42 (33.6)	43 (34.4)	0.894
Ever received COVID-19 Vaccines, n (%)	191 (76.4)	95 (76.0)	96 (76.8)	0.882
Ever received COVID-19 Booter, n (%)	95 (38.0)	49 (39.2)	46 (36.8)	0.696
Perception of COVID-19 Vaccines, mean (SD)	3.02 (0.72)	3.05 (0.69)	2.98 (0.76)	0.390

Section 2: Test the effectiveness of an onsite PoC SARS-CoV-2 intervention.

H1: O-PoC (vs. SoC) will result in a greater proportion of tests performed and results received.

a. **Associations between study arms (O-POC vs. Standard of Care) and measures of COVID-19 tests between enrollment - months 3.**

As of 06/17/2024, we have months 3 COVID-19 testing data for 170 participants (O-POC=90, Standard of care=80). Table 2 presents Chi-square tests (or Fisher exact tests) for the associations between study arms (O-POC vs. Standard of Care) and measures of COVID-19 tests between enrollment - month 3. As compared to the Standard of Care arm, participants in the O-POC arm had significantly higher rate of test results received and higher PCR-test rate, but lower antigen test rate. The differences in overall COVID-19 test rate were not significant between the two arms.

Table 2. Chi-square tests (or Fisher exact tests) and t-tests for the associations between study arms (O-POC vs. Standard of Care) and measures of COVID-19 tests between enrollment - month 3.

	O-POC (N=90)	Standard of Care (N=80)	p-value
COVID-19 tests (yes/no), n (%)	84 (93.3)	68 (85.0)	0.078
COVID-19 test results received (yes/no), n (%)	84 (93.3)	64 (82.5)	0.029

PCR-tests (yes/no), n (%)	81 (90.0)	38 (47.5)	<0.001
Antigen-tests (yes/no), n (%)	15 (16.7)	32 (40.0)	<0.001
Number of COVID-19 tests, mean (SD)	1.54 (1.12)	1.31 (1.10)	0.186
COVID-19 Vaccination Status Changed	13 (14.4)	6 (7.5)	0.152
Perception of COVID-19 Vaccines, mean (SD)	3.15 (0.65)	2.97 (0.67)	0.081

Table 3 presents results of logistic regression analyses of study arms (O-POC vs. Standard of Care) as related to measures of COVID-19 tests between enrollment – month 3 with/without controlling for baseline characteristics and history of COVID-19 tests and vaccinations. As compared to the Standard of Care arm, participants in the O-POC arm had significantly higher likelihood of receiving at least a test result and having PCR-test, but lower likelihood of having antigen test. Overall, the differences in likelihood of having at least a COVID-19 test were not significant between the two arms (with or without controlling for the covariates).

Table 3. Logistic regressions of study arms (O-POC vs. Standard of Care) as related to measures of COVID-19 tests between enrollment – month 3.

	**Bivariate Logistic Regression**		**Multiple Logistic Regression** [a]	
Dependent Variables	OR (95% CI)	p-value	AOR (95% CI)	p-value
COVID-19 tests (yes/no)	2.47 (0.88 – 6.93)	0.086	2.19 (0.73 – 6.51)	0.160
COVID-19 test results received (yes/no)	2.97 (1.08 – 8.15)	0.035	2.85 (1.00 – 8.11)	0.050
PCR-tests (yes/no)	9.95 (4.40 – 22.51)	<0.001	10.52 (4.55 – 24.34)	<0.001
Antigen-tests (yes/no)	0.30 (0.15 – 0.61)	0.001	0.24 (0.11 – 0.51)	<0.001

Note: [a] Age, gender, ethnicity, living situation, ever-tested, ever-vaccinated, and number of days incarcerated in the past 30 days before enrollment were controlled in the multiple logistic regressions.

Table 4 presents results of Poisson regression analyses of study arms (O-POC vs. Standard of Care) as related to number of COVID-19 tests between enrollment – month 3 with/without controlling for baseline characteristics and history of COVID-19 tests and vaccinations. As compared to the Standard of Care arm, participants in the O-POC arm had greater number COVID-19 tests, but not statistically significant.

Table 4. Poisson regressions of study arms (O-POC vs. Standard of Care) as related to number of COVID-19 tests between enrollment – month 3.

	**Bivariate Poisson Regression**		**Multiple Poisson Regression** [a]	
Dependent Variables	IRR (95% CI)	p-value	IRR (95% CI)	p-value
Number of COVID-19 tests	1.17 (0.94 – 1.47)	0.161	1.13 (0.91 – 1.42)	0.275

Note: [a] Age, gender, ethnicity, living situation, ever-tested, ever-vaccinated, and number of days incarcerated in the past 30 days before enrollment were controlled in the multiple Poisson regressions.

b. Associations between study arms (O-POC vs. Standard of Care) and measures of COVID-19 tests between months 3-6.

As of 06/17/2024, we have months 6 COVID-19 testing data for 145 participants (O-POC=74, Standard of care=71). Table 5 presents Chi-square tests (or Fisher exact tests) for the associations between study arms (O-POC vs. Standard of Care) and measures of COVID-19 tests between months 3-6.

Table 5. Chi-square tests (or Fisher exact tests) and t-tests for the associations between study arms (O-POC vs. Standard of Care) and measures of COVID-19 tests between months 3-6.

	O-POC (N=74)	Standard of Care (N=71)	p-value

COVID-19 tests (yes/no), n (%)	61 (82.4)	47 (66.2)	0.025
COVID-19 test results received (yes/no), n (%)	61 (82.4)	46 (64.8)	0.016
PCR-tests (yes/no), n (%)	57 (77.0)	28 (39.4)	<0.001
Antigen-tests (yes/no), n (%)	13 (17.6)	17 (23.9)	0.343
Number of COVID-19 tests, mean (SD)	1.32 (1.25)	0.99 (0.94)	0.069
COVID-19 Vaccination Status Changed	2 (2.7)	6 (8.5)	0.160
Perception of COVID-19 Vaccines, mean (SD)	3.13 (0.65)	2.99 (0.74)	0.236

Table 6 presents results of logistic regression analyses of study arms (O-POC vs. Standard of Care) as related to measures of COVID-19 tests between months 3–5 with/without controlling for baseline characteristics and history of COVID-19 tests and vaccinations.

Table 6. Logistic regressions of study arms (O-POC vs. Standard of Care) as related to measures of COVID-19 tests between 3-6 months.

	**Bivariate Logistic Regression**		**Multiple Logistic Regression** [a]	
Dependent Variables	OR (95% CI)	p-value	AOR (95% CI)	p-value
COVID-19 tests (yes/no)	2.40 (1.10 – 5.20)	0.027	2.37 (1.05 – 5.34)	0.038
COVID-19 test results received (yes/no)	2.55 (1.18 – 5.52)	0.017	2.54 (1.13 – 5.72)	0.024
PCR-tests between 3-6 months (yes/no)	5.15 (2.50 – 10.59)	<0.001	5.15 (2.45 – 10.83)	<0.001
Antigen-tests between 3-6 months (yes/no)	0.68 (0.30 – 1.52)	0.345	0.52 (0.21 – 1.33)	0.172

Note: [a] Age, gender, ethnicity, living situation, ever-tested, ever-vaccinated, and number of days incarcerated in the past 30 days before enrollment were controlled in the multiple logistic regressions.

Table 7 presents results of Poisson regression analyses of study arms (O-POC vs. Standard of Care) as related to number of COVID-19 tests with/without controlling for baseline characteristics and history of COVID-19 tests and vaccinations.

Table 7. Poisson regressions of study arms (O-POC vs. Standard of Care) as related to number of COVID-19 tests.

	**Bivariate Poisson Regression**		**Multiple Poisson Regression** [a]	
Dependent Variables	IRR (95% CI)	p-value	IRR (95% CI)	p-value
Number of COVID-19 tests between months 3-6	1.34 (0.98 – 1.84)	0.063	1.33 (0.97 – 1.83)	0.079
Number of COVID-19 tests between enrollment – month 6	1.28 (1.03 – 1.60)	0.027	1.23 (0.99 – 1.54)	0.062

Note: [a] Age, gender, ethnicity, living situation, ever-tested, ever-vaccinated, and number of days incarcerated in the past 30 days before enrollment were controlled

H2: O-PoC (vs. SoC) will be associated with increased mitigation behaviors.

Among the 250 MOSAIC participants, so far, mitigation behaviors were assessed for 249 participants. On a scale of 1-100, mean (SD) masking, social distancing, and hand washing were 52.6 (36.7), 54.9 (35.1), and 73.4 (29.9), respectively. The three measures were significantly correlated with each other (r=0.45 -0.80, p<0.001).

Table 8. Mean and Standard Deviation of measures of mitigation behaviors by study arms.

	O-POC	Standard of Care
Masking, mean (SD)	57.2 (36.4)	47.6 (36.4)
Social distancing, mean (SD)	57.7 (34.8)	51.8 (35.2)
Hand washing, mean (SD)	74.9 (28.9)	71.8 (30.8)

Table 9 presents results of GEE models for the associations between study arms (O-POC vs. Standard of Care) and mitigation behaviors over time. As compared to the Standard of Care arm, participants in the O-POC arm had higher level of mitigation behaviors with masking and overall mitigation behavior being statistically significant.

Table 9. GEE models of study arms (O-POC vs. Standard of Care) as related to mitigation behaviors.

	**Bivariate GEE models**		**Multiple GEE models** [a]	
Dependent Variables	beta (95% CI)	p-value	beta (95% CI)	p-value
Social distancing	6.76 (-0.80 – 14.21)	0.080	6.30 (-1.32 – 13.91)	0.105
**Masking**	**10.00 (1.83 – 18.17)**	**0.016**	**9.60 (1.39 – 17.81)**	**0.022**
Hand washing	5.17 (-1.32 – 11.66)	0.119	4.78 (-1.62 – 11.18)	0.143
**Overall mitigation behaviors**	**7.36 (0.78 – 13.94)**	**0.028**	**6.94 (0.32 – 13.56)**	**0.040**
**Masking (weighted)** [b]	**9.55 (1.43 – 17.67)**	**0.021**	**8.89 (0.80 – 16.99)**	**0.031**

Note: [a] Age, gender, ethnicity, living situation, ever-tested, ever-vaccinated, and number of days incarcerated in the past 30 days before enrollment were controlled; [b] masking (weighted) was calculated by accounting for each reported situation and frequency.

c. Testing data from the Fortune (Sharepoint Data)

Table 10. Chi-square tests of Measures of COVID-19 tests by study arms.

	**Total**	**OPOC (on site)**	**SOC (off site)**	**P-Value**
**Tested at 1st visit**	250	98/125 (78%)	62/125 (50%)	<0.001
**Tested at 2nd visit**	170	61/90 (68%)	29/80 (36%)	<0.001
**Tested at 3rd visit**	145	43/74 (58%)	10/71 (14%)	<0.001
**Tested at 4th visit**	110	25/59 (42%)	11/51 (22%)	0.020
**Tested at 5th visit**	96	24/51 (47%)	5/45 (11%)	0.001

Note: Denominators for tested 1st-5th visits were the baseline, m3, m6, m9, and m12 samples, respectively.

Table 11. Chi-square tests of Measures of COVID-19 tests by study arms.

	**Total**	**OPOC (on site)**	**SOC (off site)**	**P-Value**
**Tested at 1st visit**	250	98/125 (78%)	62/125 (50%)	<0.001
**Tested at 2nd visit**	250	63/125 (50%)	31/125 (25%)	<0.001
**Tested at 3rd visit**	250	46/125 (37%)	10/125 (8%)	<0.001
**Tested at 4th visit**	250	30/125 (24%)	12/125 (9.6%)	0.002
**Tested at 5th visit**	250	25/125 (20%)	6/125 (5%)	0.001

Note: Denominators for tested 1st-5th visits were the baseline sample.
